# Re-appraising assays on permeabilized blood cancer cells testing venetoclax or other BH3 mimetic agents selectively targeting pro-survival BCL2 proteins

**DOI:** 10.1038/s41418-025-01487-7

**Published:** 2025-04-09

**Authors:** Jia-Nan Gong, Tirta M. Djajawi, Donia M. Moujalled, Giovanna Pomilio, Tiffany Khong, Li-Ping Zhang, Pasquale L. Fedele, Michael S. Low, Mary Ann Anderson, Christopher D. Riffkin, Christine A. White, Ping Lan, Guillaume Lessene, Marco J. Herold, Andreas Strasser, Andrew Spencer, George Grigoriadis, Andrew H. Wei, Mark F. van Delft, Andrew W. Roberts, David C. S. Huang

**Affiliations:** 1 Walter and Eliza Hall Institute of Medical Research, Melbourne, VIC Australia; 2 Departments of Medical Biology, University of Melbourne, Melbourne, VIC Australia; 3 NHC Key Laboratory of Human Disease Comparative Medicine, The Institute of Laboratory Animal Sciences, Chinese Academy of Medical Sciences & Peking Union Medical College, National Human Diseases Animal Model Resource Center, National Center of Technology Innovation for Animal Model, Beijing, China; 4 Australian Centre for Blood Diseases, Alfred Health—Monash University, Melbourne, VIC Australia; 5 Department of Clinical Haematology, The Alfred Hospital, Melbourne, VIC Australia; 6 Malignant Haematology and Stem Cell Transplantation, The Alfred Hospital, Melbourne, VIC Australia; 7 Department of Haematology, Monash Health, Clayton, VIC Australia; 8 School of Clinical Sciences at Monash Health, Monash University, Clayton, VIC Australia; 9 Clinical Haematology, The Royal Melbourne Hospital and Peter MacCallum Cancer Centre, Melbourne, VIC Australia; 10 Department of Pharmacology and Therapeutics, University of Melbourne, Melbourne, VIC Australia; 11 Present Address: Olivia Newton-John Cancer Research Institute, Heidelberg, VIC Australia; 12 Present Address: School of Cancer Medicine, La Trobe University, Bundoora, VIC Australia; 13 Present Address: oNKo-Innate, Melbourne, VIC Australia; 14 Present Address: Institute for Advanced and Applied Chemical Synthesis, Jinan University, Jinan, China

**Keywords:** Molecular biology, Translational research

## Abstract

BH3 mimetic drugs that selectively target the pro-survival BCL2 proteins are highly promising for cancer treatment, most notably for treating blood cancers. Venetoclax, which inhibits BCL2, is now approved for treating chronic lymphocytic leukemia (CLL) and acute myeloid leukemia (AML). Preferably, robust and validated assays would identify patients most likely to benefit from therapy with venetoclax itself or with inhibitors of other pro-survival proteins. A sophisticated method that has been developed is the BH3 profiling assay. In this assay, permeabilized, instead of intact, cells are treated for a few hours with inhibitors of the pro-survival BCL2 proteins, and the resultant mitochondrial depolarization measured. Sensitivity to a specific inhibitor (e.g., venetoclax or other BH3 mimetics) is then used to infer the reliance of a tumor (e.g., CLL) on one or more pro-survival BCL2 proteins. However, we found that this methodology cannot reliably identify such dependencies. In part, this is because almost all cells express multiple pro-survival BCL2 proteins that restrain BAX and BAK which must be inhibited before mitochondrial depolarization and apoptosis can proceed. Using genetic and pharmacological tools across multiple cell line models of blood cancer, we demonstrated that selective BCL2 inhibitors have important flow-on effects that includes the redistribution of BH3-only proteins to ancillary pro-survival proteins not directly engaged by the inhibitor. These secondary effects, critical to the biological action of selective inhibitors, were not accurately recapitulated in permeabilized cells, probably due to the limited time frame possible in such assays or the altered biophysical conditions when cells are permeabilized. While we could consistently define the sensitivity of a tumor cell to a particular BH3 mimetic drugs using intact cells, this was not reliable with permeabilized cells. These studies emphasize the need to carefully evaluate assays on permeabilized cells undertaken with inhibitors of the pro-survival BCL2 proteins.

## Introduction

The uptake of venetoclax into routine practice highlights the clinical value of targeting the pro-survival BCL2 proteins in hematological malignancies [1]. As a single agent, venetoclax induces high rates of remission in chronic lymphocytic leukemia (CLL) and displays impressive activity across a range of hematological malignancies. Aside from CLL, venetoclax also has regulatory approvals for treating acute myeloid leukemia (AML).

BH3 mimetic drugs, like venetoclax, mimic the pro-apoptotic BH3-only proteins (e.g., BIM, BAD, NOXA) acting to block one or more of the pro-survival BCL2 proteins thereby allowing the essential apoptosis mediators BAX and BAK to drive mitochondrial outer membrane permeabilization (MOMP) and, thus, trigger apoptosis [2–4]. Given the clinical efficacy of inhibiting BCL2 with venetoclax and considering ongoing efforts to target the other pro-survival proteins [3, 4], an important consideration is to identify which patients will derive most benefit from a BH3 mimetic drug targeting BCL2 itself or ones targeting other pro-survival BCL2 proteins.

In CLL, previous studies had implicated the essential role of BCL2 for their survival [5–11], a conclusion validated by the clinical experience with venetoclax [12]. Beyond CLL, the dependence of other tumor types on a particular pro-survival protein or combination of them is less clear. “BH3 profiling assays” exploit measurement of mitochondrial depolarization upon adding an inhibitor of the pro-survival BCL2 proteins (e.g., venetoclax) to permeabilized cells instead of conventional cell viability assays undertaken with intact cells in the laboratory. BH3 profiling has been utilized for defining the dependence of tumor cells on a specific pro-survival BCL2 protein or a combination of them [11, 13–21]. Interestingly, a recent study has reported that ex vivo venetoclax sensitivity predicts clinical response in AML to the drug [22].

We observed that relatively high concentrations of venetoclax were required for the mitochondrial depolarization of permeabilized CLL cells. Surprisingly, the activity of venetoclax in such BH3 profiling assays could not be readily distinguished from BH3 mimetics targeting other pro-survival BCL2 proteins despite these inhibitors being substantially less active in intact CLL cells. Using a range of genetic and pharmacological approaches, we identified previously under-appreciated limitations with BH3 profiling assays and highlight caution with interpreting results from such assays.

## Results

### BH3 profiling assays on permeabilized CLL cells cannot readily distinguish the impact of targeting BCL2 with venetoclax from that of BH3 mimetic compounds targeting other pro-survival relatives of BCL2

**Fig. 1:**
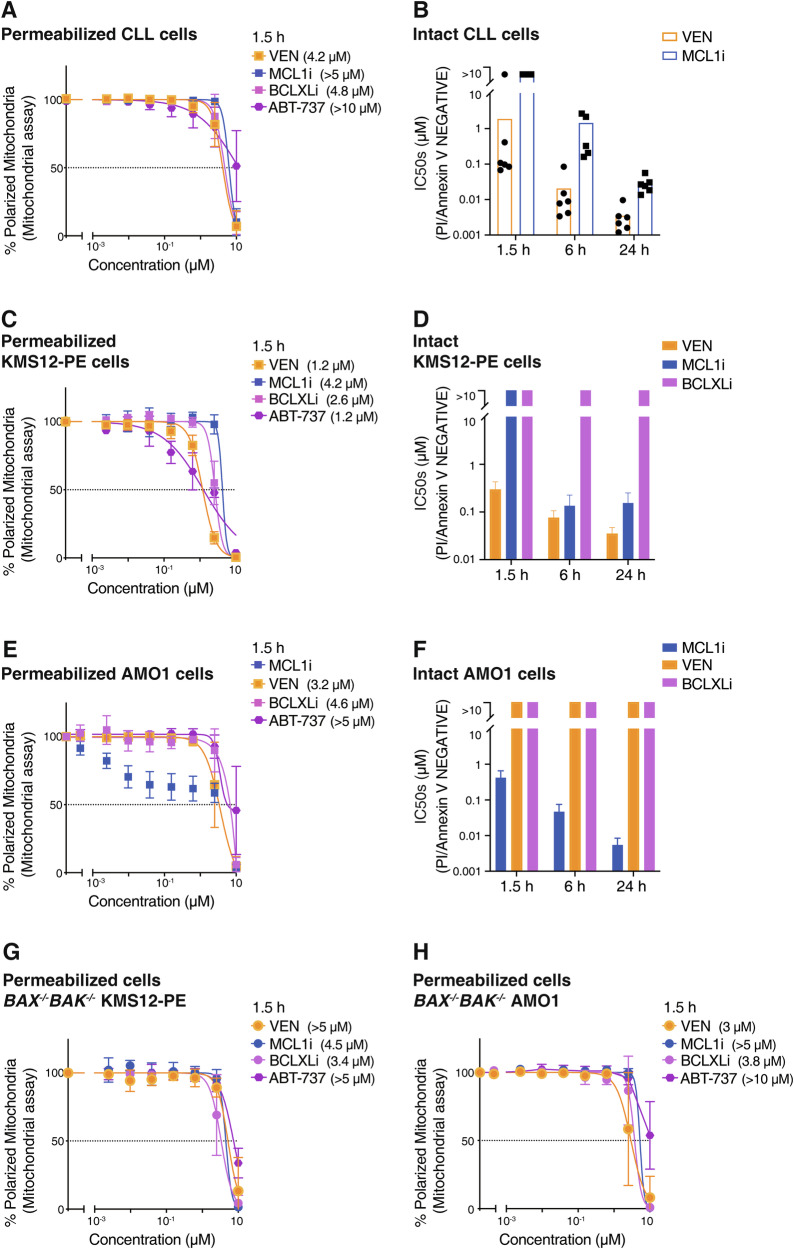
Induction of mitochondrial outer membrane permeabilization (MOMP) when permeabilized cells are directly treated with BH3 mimetic compounds. **A** Efficacy of various BH3 mimetic compounds at causing MOMP when added directly to permeabilized CLL cells. Freshly isolated primary CLL samples (*n* = 6) were permeabilized and then treated with 0–10 µM of the indicated BH3 mimetic (binding specificity summarized in Fig. S1A) to selectively target BCL2, MCL1 or BCLXL, or with ABT-737 which targets BCL2, BCLXL and BCLW, and mitochondrial depolarization determined 1.5 h later. Data represents mean IC_50_ ± SD of the 6 samples; experiment performed once per sample. **B** Sensitivity of CLL cells to BH3 mimetics. Intact CLL cells, identical to those permeabilized in (**A**), were treated for 1.5–24 h with 0–10 µM of the indicated BH3 mimetic. Viable cells (PI^-ve^/Annexin V^-ve^) were identified by flow cytometric analysis and the resulting IC_50_ calculated. Each data point represents a patient sample studied once; 6 patient samples were studied. **C** Similar BH3 profiling assays to those in (**A**) were undertaken with permeabilized KMS-12-PE cells, a multiple myeloma cell line. (**D**) As in (**B**), cell viability assays were undertaken with intact KMS-12-PE cells treated with the indicated BH3 mimetic (the dose responses are shown in Figs. S1B-C). Identical sets of experiments were undertaken with (**E**) permeabilized AMO1 myeloma cells or (**F**) intact cells (dose responses shown in Figs. S1H-I). **G** At high doses, venetoclax or other BH3 mimetics could cause non-specific mitochondrial damage. Mitochondrial assays were undertaken after treating permeabilized *BAX*^*–/–*^*BAK*^*–/–*^ KMS-12-PE cells with the indicated BH3 mimetic for 1.5 h. **H** Similar assays to those in (**G**) were undertaken with permeabilized *BAX/BAK*-deficient AMO1 cells. The data in (**C**)–(**H**) represents the means ± SD of ≥3 independent experiments; IC_50_ indicated in parentheses. See also Fig. S1.

As CLL cells have an established dependency on BCL2, we anticipated that mitochondria in permeabilized CLL cells should be rapidly depolarized by venetoclax treatment in BH3 profiling assays. On the other hand, inhibitors of MCL1 (S63845 [23]; “MCL1i”) or BCLXL (A-1331852 [24]; “BCLXLi”) should be much less active than venetoclax (Fig. S1A). As we and others previously reported [11, 25, 26], BH3 mimetics targeting BCL2 could trigger MOMP in permeabilized CLL cells, but a high concentration of the drug was required (IC_50_ > 1 µM; Fig. 1A). Surprisingly, we found that the dose response of permeabilized CLL cells to venetoclax in this assay could not be readily distinguished from that of the responses to drugs inhibiting MCL1, BCLXL, or to ABT-737 which targets BCL2, BCLXL and BCLW.

Intact (non-permeabilized) CLL cells were sensitive to venetoclax (IC_50_ ~ 1 μM @ 1.5 h, ~10 nM @ 24 h; Fig. 1B) as previously reported [27]. In accord with previous studies [28, 29], MCL1 inhibition showed modest activity (IC_50_ > 10 μM @ 1.5 h: ~50 nM @ 24 h), whereas BCLXL inhibition is known to be inactive on CLL [27, 30, 31]. Thus, while BCL2i was clearly the most active BH3 mimetic when tested on intact CLL cells (Fig. 1B), their activity could not be easily distinguished from each other when tested on permeabilized CLL cells (Fig. 1A).

We sought to understand why the potency of venetoclax was clearly distinguishable from other BH3 mimetics when tested on intact CLL cells (Fig. 1B), but not when tested on identical cells after permeabilization (Fig. 1A). To explore these discrepancies, we undertook similar experiments using immortalized blood cancer cell lines that are widely used for testing BH3 mimetic compounds.

### A range of BH3 mimetic compounds have indistinguishable activity when tested on various permeabilized cancer cell lines

Akin to results obtained using permeabilized CLL cells, μM concentrations of diverse BH3 mimetics were also required to induce MOMP when added to permeabilized KMS-12-PE multiple myeloma (MM) cells (Fig. 1C). By comparison, venetoclax was clearly most active against intact KMS-12-PE cells, with MCL1i being moderately active and BCLxLi inactive (Fig. 1D, S1B, S1C). Similar observations were made after testing various BH3 mimetics on other cell lines. While the compounds showed a range of activity on intact cells, for example on intact RS4;11 acute lymphoblastic leukemia (ALL) cells (venetoclax>MCL1i; Fig. S1D) or MV4-11 acute myeloid leukemia (AML) cells (venetoclax>MCL1i; Fig. S1F), comparable μM amounts of the same BH3 mimetics were required to cause MOMP when the same cells were permeabilized (Figs. S1E, S1G). In another myeloma cell line AMO1, MCL1i showed some activity on permeabilized cells (Fig. 1E) but, when tested on intact cells, it was clearly much more active than any of the other inhibitors tested (Fig. 1F, S1H, S1I).

Strikingly, the induction of MOMP observed when μM concentrations of BH3 mimetics were added to permeabilized cells appeared to be mediated non-specifically since their activities were only minimally attenuated when tested on isogenic cells lacking BAX/BAK, the essential mediators of MOMP [32, 33] (Figs. 1G, H, S1L). In KMS-12-PE cells, for example, there was only minor attenuation in the ability of venetoclax to directly cause mitochondrial depolarization in isogenic cells lacking BAX/BAK (IC_50_ > 5 μM; Fig. 1G) compared to parental cells (IC_50_ ~ 1.2 μM; Fig. 1C). Similarly, the action of various BH3 mimetics on OPM2 mitochondria was minimally affected by deleting BAX/BAK (compare Fig. S1L with Fig. S1J). The only exception was in the MCL1-dependent AMO1 cells where loss of BAX/BAK blunted their response to MCL1i (compare Fig. 1H with 1E), suggesting that modest response of permeabilized AMO1 cells to MCL1i was BAX/BAK-mediated and, hence, specific.

For all the BH3 mimetics we tested, high (μM) concentrations were required to induce MOMP in permeabilized cells (Figs. 1A, C, S1E, S1G, S1J) and the depolarization was, in most of the cell lines we studied, not principally MOMP driven specifically by BAX/BAK (Fig. 1G, S1L). To resolve this conundrum, we next examined a range of BH3 peptides instead of BH3 mimetic compounds (Fig. S2A). These have been widely used in BH3 profiling assays at concentrations, often varying, of up to 100 μM [11, 13, 17, 18, 20, 34].

### A peptide from the BH3 domain of pro-apoptotic BIM potently and specifically induces MOMP when added to permeabilized cells

As their binding to the pro-survival BCL2 proteins are well documented, such peptides have been used as surrogates for determining the dependence of tumor cells on specific pro-survival BCL2 proteins in BH3 profiling assays. For example, the selective binding of BH3^BAD^ to BCL2 (as well as BCLXL and BCLW) [35, 36] has been used to infer dependence on BCL2. On the other hand, MCL1-dependence [37, 38] has been deduced from sensitivity to BH3^BIM2A^ or BH3^MS1^. These peptides are not cell penetrant and thus, could not be tested on intact cells.

We evaluated these peptidyl reagents at up to 160 μM on a selection of cells lines after permeabilization. Some (KMS-12-PE, Fig. 1D; RS4;11, Fig. S1D; MV4-11, Fig. S1C) are most sensitive to venetoclax, whereas AMO1 (Fig. 1F) and OPM2 (Fig. S1K) are most sensitive to MCL1i. Where we could, we compared parental cells with their matching BAX/BAK-deficient counterparts to assess if the peptides acted through BAX/BAK-driven MOMP.

**Fig. 2:**
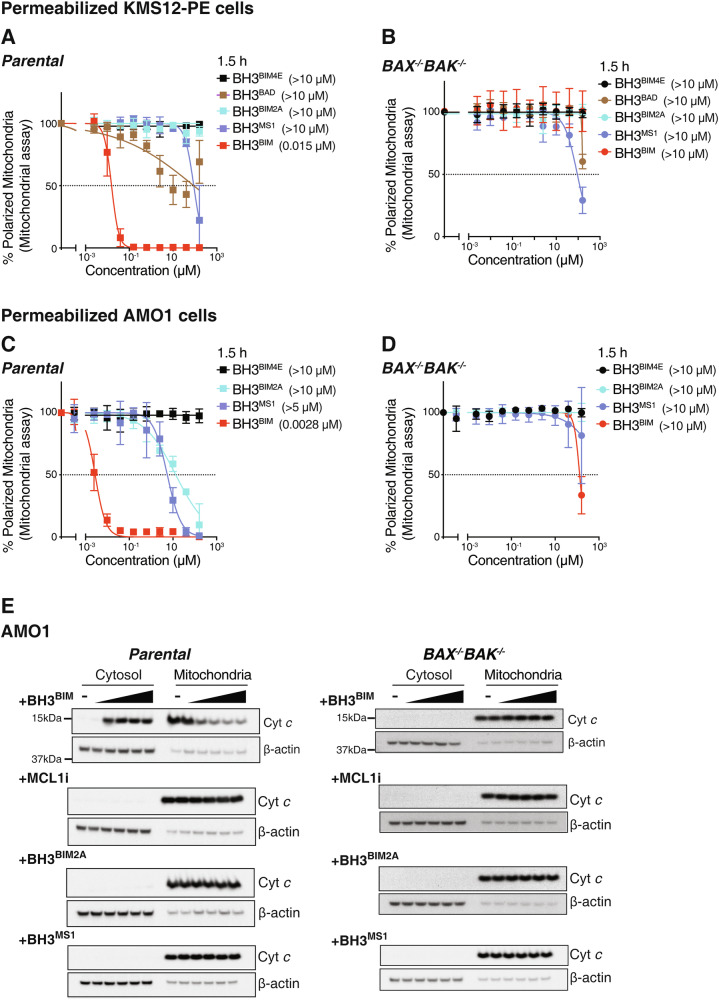
Efficacy of BH3 domain peptides at inducing MOMP when added to permeabilized cells. Mitochondrial assays were performed on permeabilized parental KMS-12-PE cells (**A**) or their isogenic *BAX*^-/-^*BAK*^-/-^ counterparts (**B**) after treatment for 1.5 h with 0–160 µM of the indicated BH3 peptide; the binding specificities of peptides are shown in Fig. S2A. Identical mitochondrial assays were undertaken with permeabilized parental (**C**) or *BAX*^–/–^*BAK*^–/–^ (**D**) AMO1 cells. (**E**) A peptide from the BH3 domain of BIM (BH3^BIM^) causes BAX/BAK-dependent release of Cytochrome *c*. Permeabilized AMO1 cells of the indicated genotype were treated with 0–10 µM of the BH3^BIM^ peptide for 1 h, fractionated and blotted for Cytochrome *c*; loading control: β-actin. The blots are representative of 2 independent experiments; original uncropped immunoblots are provided with Supplemental materials. Data from treatment with other BH3 reagents is shown in Fig. S2F. **F** BH3^BIM^ peptide induces marked mitochondrial depolarization when added to permeabilized CLL cells. These permeabilized cells were treated with the indicated peptide and mitochondrial depolarization determined 1.5 h later. Data represents mean IC_50_ ± SD of the 6 patient samples studied; the experiment was performed once per sample. The data in (**A**)–(**D**) represents the means ± SD of ≥3 independent experiments. IC_50_ indicated in parentheses. See also Fig. S2.

As expected from previous studies, a peptide derived from the BH3 domain of pro-apoptotic BIM (BH3^BIM^) [36, 39, 40] showed potent activity in all the permeabilized cell lines tested (IC_50_ < 50 nM; Figs. 2A, C, S2B, S2C, S2D). In the “BCL2-dependent” line KMS-12-PE, BH3^BAD^ was active but it was much more modest than BH3^BIM^ (Fig. 2A); in two other venetoclax-sensitive lines (RS4;11 Fig. S2B and MV4-11 Fig. S2C), there was barely any activity with the “BCL2-selective ligand” BH3^BAD^ at the very highest peptide concentrations used (160 μM) even these cell lines are more sensitive to venetoclax (Figs. S1D, S1F). Both BH3^BIM2A^ and BH3^MS1^ had some activity on AMO1 (Fig. 2C) and OPM2 (Fig. S2D), cell lines that are MCL1i-sensitive.

Except at the very highest concentrations tested, most of the peptides showed specificity in their mode-of-action since MOMP induction was abrogated when BAX/BAK were deleted (Figs. 2B, D, S2E). Interestingly, lack of specificity observed with some peptides was seen at peptide concentrations >100 μM (Figs. 2B, D, S2E), higher than observed with the BH3 mimetic compounds (~10 μM; Figs. 1G, H, S1L).

Notably, all the reagents that selectively bind to sub-sets of the pro-survival BCL2 proteins were significantly less active than BH3^BIM^ when assayed on the same cells (e.g., BH3^BAD^ vs BH3^BIM^ in permeabilized KMS-12-PE cells Fig. 2A; BH3^BIM2A^/BH3^MS1^ vs BH3^BIM^ in permeabilized AMO1 cells Fig. 2C). This observation was confirmed by examining BAX/BAK-driven release of mitochondrial Cytochrome *c* into the cytosol of permeabilized AMO1 cells where BH3^BIM^ was highly effective but the other reagents selectively targeting MCL1 were not (Fig. 2E), even though this cell line is exquisitely sensitive to MCL1 inhibition (Fig. 1F) [41].

Finally, BH3^BIM^ also potently induced MOMP when added to permeabilized CLL cells (Fig. S2F), but none of the other peptides, including BH3^BAD^ which targets BCL2, could when they were tested at up to 10 μM. With these selective peptides, some activity at higher (50 μM) concentrations have been previously reported [20, 34]. Interestingly, others have noted that such selective peptides are less active than BH3^BIM^ when treating permeabilized CLL cells [11, 31].

### BCL2 limits the ability of an MCL1 inhibitor to induce MOMP in permeabilized AMO1 cells

As BH3^BIM^ is highly effective at specifically inducing BAX/BAK-driven MOMP in the assays on permeabilized cells (Fig. 2), we next considered which of its properties might be lacking in the other inhibitors to explain why these are much less active. Potentially, BH3^BIM^ could directly activate BAX/BAK, but this is usually evident at high (µM) peptide concentrations [36, 39, 42]. Alternatively, BH3^BIM^ may be more active because it binds to all the pro-survival BCL2 proteins [35], whereas the other inhibitors tested, either compounds (Fig. S1A) or peptides (Fig. S2A), only act on selective targets. As most cells express multiple pro-survival proteins, we postulate that in these assays on permeabilized cells treated with the selective agents (e.g. venetoclax or BIM^BAD^ on CLL cells; Fig. 1A, S2F), MOMP could not efficiently proceed because of the actions of other, ancillary pro-survival proteins (e.g., MCL1 in permeabilized CLL cells) even when the principal pro-survival protein they harbor (e.g., BCL2 in CLL leukemic cells) was inhibited. Interestingly, it was previously shown in permeabilized CLL cells that BIM^BAD^ (to target BCL2) was more effective when combined with BH3^MS1^ (to target MCL1) [31].

**Fig. 3:**
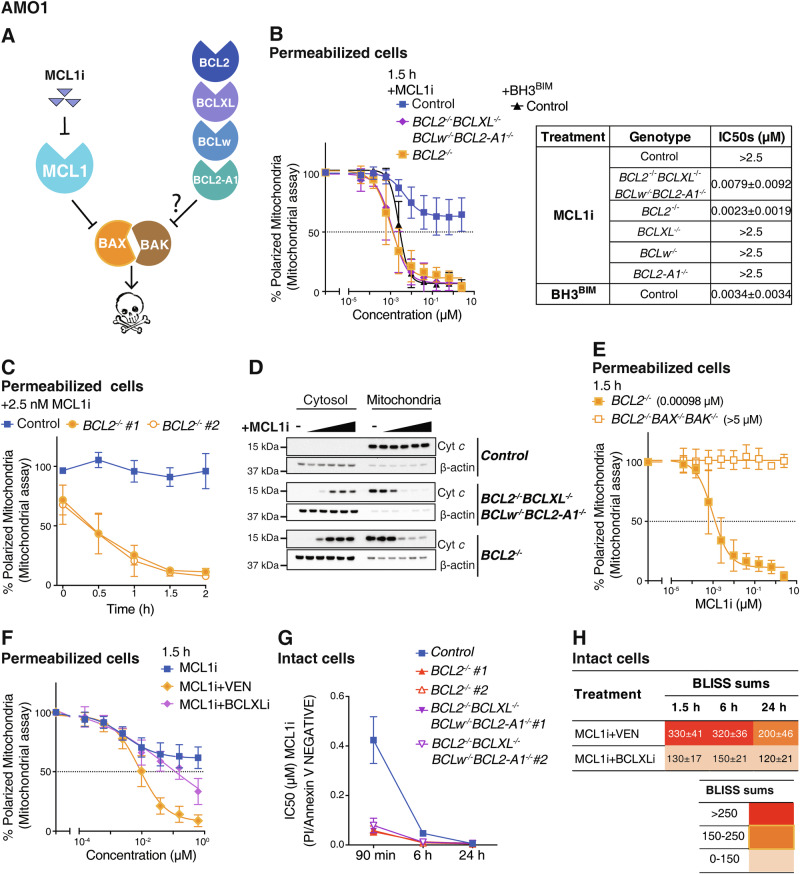
In permeabilized AMO1 cells, BCL2 limits the ability of an MCL1 inhibitor to directly cause MOMP. **A** In AMO1 cells, we postulate that ancillary pro-survival proteins (BCL2, BCLXL, BCLW and/or BCL2-A1/BFL1) could limit the impact of inhibiting the key survival factor MCL1 in this cell line. **B** BCL2 limits the ability of MCL1i to damage the mitochondria in permeabilized AMO1 cells. On the left, mitochondrial assays were performed after MCL1i treatment of permeabilized AMO1 cells transduced with the empty vector or vectors expressing guide RNAs to target one or more pro-survival *BCL2* family member. Their sensitivity (mean IC_50_ ± SD) is summarized on the right (the primary data is shown in Fig. S3A). 2 independent clones of each genotype were assayed in ≥ 3 experiments. **C** Time course experiment after treating permeabilized AMO1 cells of the indicated genotypes with a very low dose (2.5 nM) of an MCL1 inhibitor. **D** When *BCL2* is deleted, targeting MCL1 causes efficient Cytochrome *c* release from permeabilized AMO1 cells. Permeabilized AMO1 cells of the indicated genotypes were treated with 0–2.5 µM MCL1i for 1 h, fractionated and then blotted for the indicated proteins. The blots shown are representative of 2 independent experiments; loading control: β-actin. Original uncropped immunoblots are provided with Supplementary materials. **E** Deletion of *BCL2* markedly sensitizes permeabilized AMO1 cells to MCL1i, but this is fully abrogated if *BAX* and *BAK* are co-deleted. **F** Co-targeting BCL2 markedly sensitizes permeabilized AMO1 cells to MCL1i. Response of mitochondria isolated from AMO1 cells to the indicated BH3 mimetic, either alone or in equimolar combinations (1:1). (see also Fig. S3C). **G** The ability of MCL1i to induce cell death in intact AMO1 cells was enhanced in the absence of *BCL2*. Sensitivity of intact AMO1 cells of the indicated genotypes after incubation with MCL1i for 1.5–24 h. **H** Sensitization of intact AMO1 cells to MCL1i by co-targeting BCL2. The viability (PI^-ve^/Annexin V^-ve^) of AMO1 cells treated with MCL1i with the co-addition of venetoclax or BCLXLi was determined as in (**G**). In the heatmaps, the Bliss sums are shown. Briefly, the predicted additive effect was calculated using the Bliss model of fractional independence and subtracted from the actual measured combinatorial effect to generate the Bliss scores of two compounds [56]; see Table S3 for Bliss sum calculation. Values > 150 indicate substantial synergy [57, 58]. Data in (**B**), (**C**) and (**E**)–(**H**) represents the means ± SD of ≥ 3 independent experiments. See also Fig. S3.

To formally test this idea, we focused on AMO1, a cell line that is highly sensitive to MCL1i (Fig. 1F). In our proposed scenario, BH3^BIM^ could efficiently drive MOMP in permeabilized AMO1 cells (Fig. 2C) because of its ability to target MCL1, the dominant survival factor in these cells, as well as all the other pro-survival proteins (BCL2, BCLxL, BCLw, BCL2-A1) present on mitochondria (Fig. 3A). MCL1i and the MCL1-selective peptides (BH3^BIM2A^, BH3^MS1^), on the other hand, could not efficiently provoke MOMP on their own, due to several other pro-survival proteins restraining BAX/BAK activation. If this model is correct, we anticipate that MCL1i or the MCL1-selective peptides should be much more active at causing mitochondrial depolarization when all other pro-survival proteins were genetically deleted from permeabilized AMO1 cells.

While MCL1i alone displayed limited activity on permeabilized AMO1 cells (Fig. 1E), its efficacy was markedly enhanced when all the other pro-survival genes were deleted by CRISPR/Cas9 (IC_50_ < 10 nM; Fig. 3B, S3A). Deleting each of the other pro-survival proteins pinpointed the importance of BCL2 as deleting *BCL2* alone markedly sensitized AMO1 mitochondria to MCL1 inhibition. Remarkably, the response of *BCL2*^–/–^ AMO1 cells (IC_50_ 2.3 nM) to MCL1i approached that seen with BH3^BIM^ on wild-type cells (IC_50_ 7.9 nM; Figs. 3B, C, S3A), observations confirmed in orthogonal Cytochrome *c* release assays (Fig. 3D). Consistent with the sensitization to MCL1i when *BCL2* was deleted, the activity of an MCL1-slective peptide, BH3^BIM2A^, was markedly enhanced when *BCL2* was deleted (Fig. S3B). Importantly, deleting *BAX*/*BAK* from *BCL2*^–/–^ AMO1 cells resulted in their mitochondria being unresponsive to MCL1i, confirming the specificity of the observed effects (Fig. 3E, S3C).

Consistent with our data with genetically targeting *BCL2*, which revealed its role ancillary to that of MCL1 in permeabilized AMO1 cells, we found that pharmacologically co-inhibiting BCL2, but not BCLxL, significantly enhanced the efficacy of MCL1i on these cells (Fig. 3F, S3C). As expected, intact BCL2-deficient AMO1 cells are sensitized to MCL1i (Fig. 3G) and, likewise, the impact of MCL1i was enhanced by co-treating parental AMO1 cells with venetoclax (Fig. 3H).

Taken together, our data support the notion that BCL2 limits the impact of just inhibiting MCL1 to cause mitochondrial depolarization of permeabilized AMO1 cells (Fig. 3A). Removing BCL2, the remaining barrier to BAX/BAK-driven MOMP when MCL1 is inhibited, significantly enhanced sensitivity of permeabilized AMO1 cells to MCL1i (Fig. 3B) or a peptide that targets MCL1 (Fig. S3B). We conclude from these mechanistic studies that an inhibitor added directly to permeabilized cells could only efficiently provoke MOMP when all the brakes to BAX/BAK activation were removed.

### Co-targeting BCL2, the ancillary pro-survival factor in AMO1 cells, enhances the efficacy of MCL1 inhibition in vivo

**Fig. 4:**
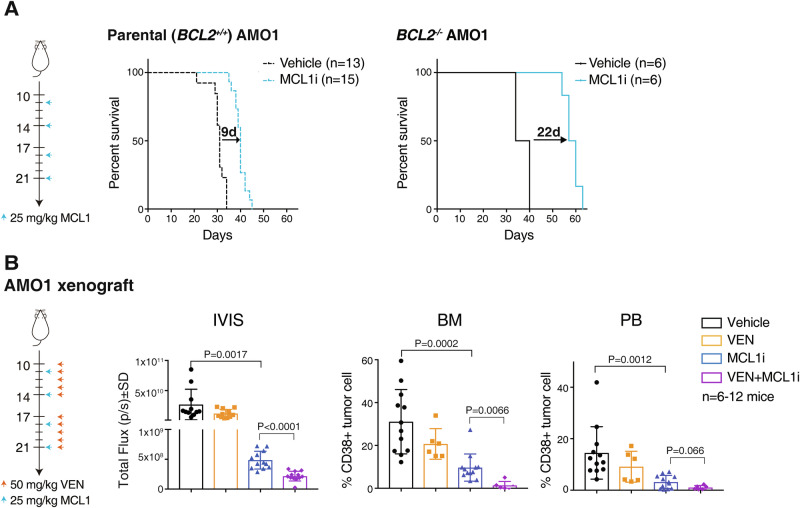
Co-targeting the ancillary survival factor improves the in vivo treatment responses of tumors. **A** Genetic deletion of *BCL2* prolonged survival of AMO1 myeloma bearing mice treated with MCL1i. NSG mice (*n* = 6–15 mice/group) were inoculated i.v. with wild-type (BCL2^+/+^; left) or BCL2^–/–^ (right) AMO1 cells. **B** Venetoclax enhances the activity of MCL1i in AMO1 myeloma bearing mice. NSG mice (*n* = 6–12 per group) were inoculated intravenously with luciferase-expressing AMO1 cells. Mice underwent baseline imaging on day 10 using IVIS and were randomized into 4 groups. The treatment schedule and doses are summarized on the left. The whole-body tumor burden (detected by IVIS in vivo imaging system) was determined on day 27, as was infiltration of tumor cells (human CD38^+ve^) in the bone morrow (BM) or peripheral blood (PB); the statistical significance between the different treatment groups was calculated using unpaired Student’s t test.

Having identified the previously unappreciated role of BCL2 in limiting the action of MCL1i in AMO1 cells (Fig. 3), initially in permeabilized cells (Figs. 3B, C, D, E, F) and then in intact cells in culture (Figs. 3G, H), we next tested whether co-targeting BCL2 could also enhance the in vivo response of AMO1 xenografts to MCL1i. While the AMO1 xenograft model responded to MCL1 inhibition, treatment outcomes were enhanced when BCL2 was co-targeted. Treating mice inoculated with AMO1 cells with MCL1i prolonged their survival (Fig. 4A) and reduced tumor burden (Fig. 4B). This was enhanced if BCL2 was co-targeted, either genetically (Fig. 4A) or pharmacologically (Fig. 4B).

### Ancillary pro-survival factors limit the full action of BH3 mimetic drugs in diverse blood cancer cell lines

Given our findings in AMO1 cells highlighting the importance of the ancillary pro-survival factors in addition to the predominant one(s) (Figs. 3, 4), we next sought to uncover whether ancillary survival factors are important for hindering the full potential of BH3 mimetic agents in other myeloma cell lines [23, 41]. Our aim was to determine if the co-targeting of ancillary survival factors identified from assays on permeabilized cells could effectively reveal the key survival factors limiting efficacy of any BH3 mimetic drug.

**Fig. 5:**
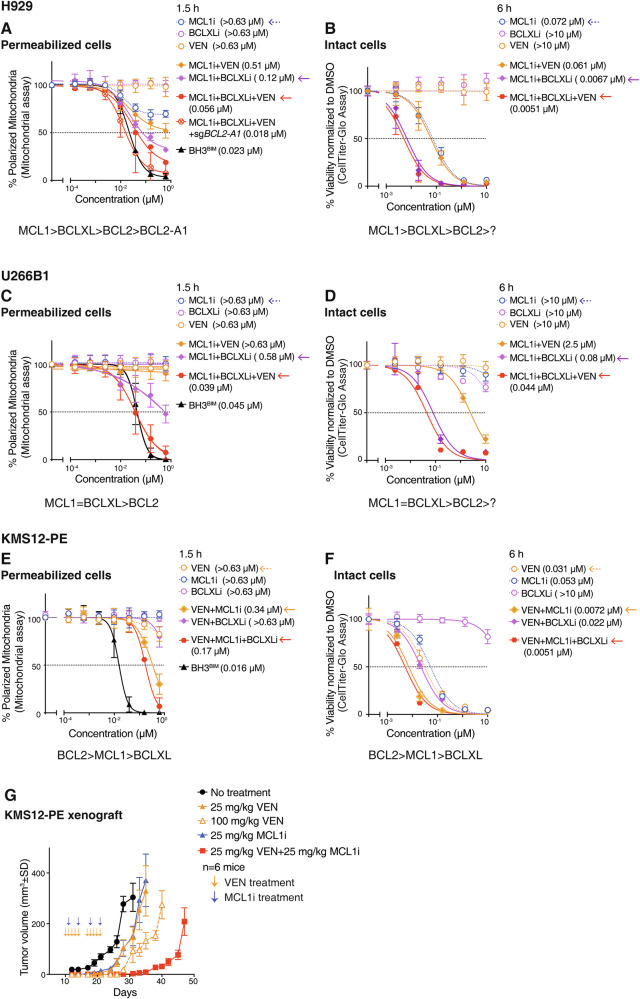
Ancillary pro-survival factors limit the full action of BH3 mimetic drugs to directly cause MOMP. **A** Efficacy of different treatments at causing MOMP when added directly to permeabilized H929 cells. BH3 profiling assays were undertaken on permeabilized H929 cells after treatment for 1.5 h with the BH3^BIM^ peptide alone or the indicated BH3 mimetics, either alone or in equimolar combinations (1:1 or 1:1:1). Also shown is the additional impact of *BCL2-A1* deletion; data shown are the average of two *BCL2-A1* knock-out clones. Sensitivity (IC_50_) to venetoclax alone, with the additional co-inhibition of MCL1, or MCL1 as well as BCLXL, are highlighted with arrows. At the bottom, the inferred dependence of permeabilized H929 cells on the different pro-survival proteins is shown. **B** Sensitivity of intact H929 cells to BH3 mimetic treatment. The viability of intact H929 cells 6 h after treatment with 0–10 µM of the indicated BH3 mimetic, either alone or in equimolar combinations (1:1 and 1:1:1) was determined. Identical sets of experiments were undertaken with permeabilized (**C**) or intact (**D**) U266B or KMS-12-PE (**E**, **F**) cells. Similar experiments to (**A**) performed on permeabilized (left) or intact (right) H929 cells. Also shown, is the additional impact of *BCL2-A1* deletion; data shown is the average of two *BCL2-A1* knock-out clones. **G** MCL1i markedly enhances the activity of venetoclax on KMS-12-PE cells in vivo. NSG mice were subcutaneously inoculated on their flanks with KMS-12-PE cells. Once the tumors were palpable, the indicated treatment was commenced. The other treatment arms are shown in Fig. S4B. Tumors were measured every 2–3 days; data represent the mean tumor volumes ± SD (n = 6 mice/group). Data in (**A**)–(**F**) represent the means ± SD of ≥ 3 independent experiments. IC_50_ indicated in parentheses. See also Fig. S4.

H929 cells are known to be MCL1-dependent [41]. While permeabilized H929 cells were slightly sensitive to MCL1i, we were able to re-capitulate the effect of BH3^BIM^ treatment when BCL2, BCLXL and BCL2-A1 were also targeted, the latter genetically (Fig. 5A). In these BH3 profiling experiments, we could thus establish a hierarchy of pro-survival factors in H929 cells with BCLXL, BCL2 and BCL2-A1 playing ancillary roles to MCL1. Consistent with this conclusion, assays with BH3 mimetics on intact H929 cells (Fig. 5B) revealed a similar trend in the importance of individual pro-survival proteins.

Permeabilized U266B1 cells (Fig. 5C) or their intact precursors (Fig. 5D) relied principally on MCL1/BCLxL for survival, with a minor contribution from BCL2. In the venetoclax-sensitive KMS-12-PE cells (Fig. 1D), co-targeting MCL1 enhanced the activity of venetoclax (Figs. 5E, F, S4A). Likewise, MCL1i potentiated the therapeutic impact of venetoclax on KMS-12-PE cells xenografted in vivo (Fig. 5G, S4B). Potentially, other pro-survival proteins (BCLW, BCL2-A1) might play a role in KMS-12-PE cells since co-targeting BCL2, MCL1 and BCLXL in this cell line when permeabilized did not re-capitulate the effect of BH3^BIM^ (Fig. 5E).

By using genetic and pharmacological co-targeting approaches in assays on permeabilized cells, we recapitulated the impact, fully in some cases, of treating permeabilized cells with BH3^BIM^ across multiple cell lines (Figs. 3B, 5A, C). Moreover, the conclusions we drew from the assays on permeabilized cells delineating the hierarchy of pro-survival BCL2 proteins were broadly consistent with studies undertaken on intact cells (Figs. 3G, H, 5B, D).

### Importance of ancillary survival factors for the action of BH3 mimetics in primary hematological cancers

Finally, we extended our studies to patient samples using combined pharmacological targeting of BCL2, BCLxL and MCL1 as a surrogate to fully define pro-survival protein dependency in primary cells. Comparisons between the efficacy of individual, pairwise or triple drug combinations allowed us to extrapolate the role of each survival factor.

**Fig. 6:**
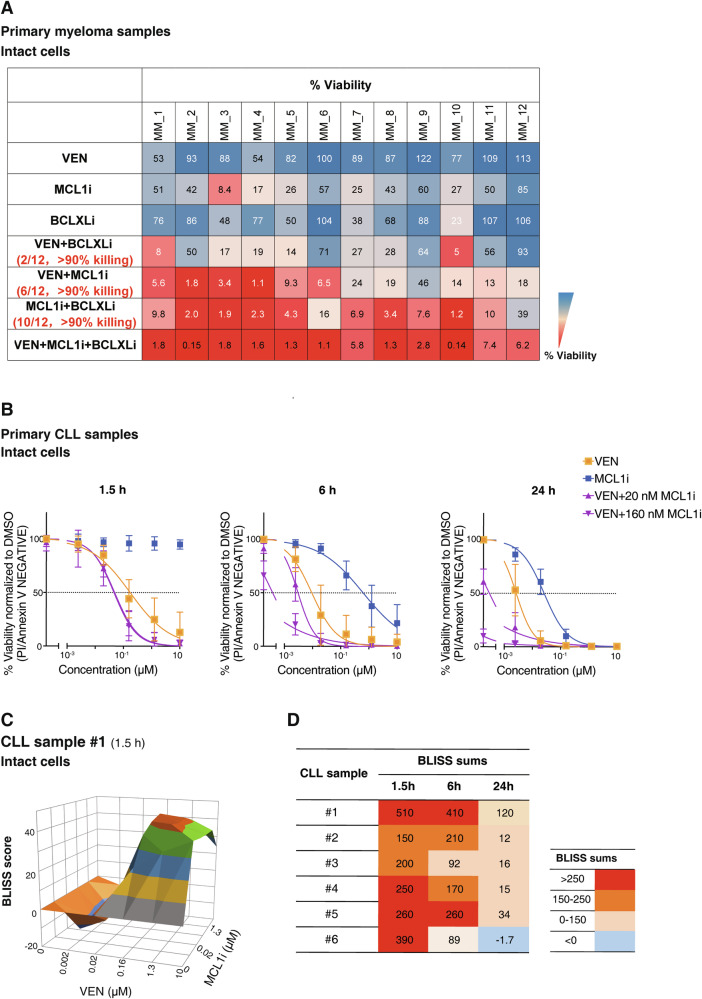
Enhanced killing of primary hematological cancer cells by co-targeting the ancillary survival factors. **A** Killing activity of BH3 mimetics in primary multiple myeloma samples when used alone or in combinations. The viability of primary patient myeloma samples (*n* = 12) after treatment for 24 h with 100 nM of the indicated BH3 mimetic, given alone or with equimolar (1:1 or 1:1:1) combinations is shown. The extent of tumor cell killing is indicated by the gradient heatmap (right). All samples were readily killed when the three BH3 mimetics were combined (bottom row). >90% killing (in red) was observed when MCL1/BCL2 were co-targeted in 6/12 samples and 10/12 samples by MCL1/BCLXL co-targeting. Data were generated from one experiment per sample. **B** Co-inhibiting MCL1 enhances killing of primary CLL cells by venetoclax. The viability (PI^-ve^/Annexin V^-ve^) of CLL patient samples (*n* = 6) treated for 1.5–24 h with venetoclax, either alone or with co-addition of MCL1i (20 nM or 160 nM), or MCL1i alone, was determined by flow cytometry. Data are shown as means ± SD derived from testing 6 patient samples. **C** Synergy between venetoclax and MCL1i in CLL cells. A representative surface plot of the Bliss sum scores in a patient sample at 1.5 h is shown. **D** Bliss sum scores of all six CLL patient samples; note the marked synergy at the earliest time point studied. See also Fig. S5.

In multiple myeloma, all samples were effectively killed by co-targeting BCL2, BCLxL and MCL1 (Fig. 6A). As expected, MCL1i was most active in multiple myeloma [41]. Interestingly, the combined effect of MCL1i and venetoclax elicited >90% cell death in 6/12 samples, 10/12 samples were just as readily killed by co-targeting MCL1/BCLxL (Fig. 6A, S5A, S5B). Thus, BCLxL appeared to be as least as important as BCL2 in restricting the full impact of MCL1i in myeloma, as suggested [43, 44]. In AML, BCL2 and MCL1 appear equally prominent (Fig. S5C) [45, 46].

To determine if these results had similar corollaries in CLL, we confirmed that CLL cells were killed by MCL1i in vitro but the extent and rate of killing was, as expected [28, 29], less than that observed with venetoclax (Figs. 1B, 6B). Notably, venetoclax-induced killing of CLL cells was dramatically enhanced even with low concentrations of MCL1i in all patient samples (Figs. 6B, C, D), suggesting that MCL1 limits the action of venetoclax against CLL cells.

Our results with patient samples extend and confirm our findings in the human cancer cell lines that secondary survival factors dampen the full impact of targeting the dominant survival factor in diverse hematological malignancies: BCL2 or BCLxL for MCL1i in myeloma (Fig. 6A) and in the case of CLL, MCL1 for venetoclax (Fig. 6B).

### Direct and indirect consequences of targeting pro-survival BCL2 proteins

**Fig. 7:**
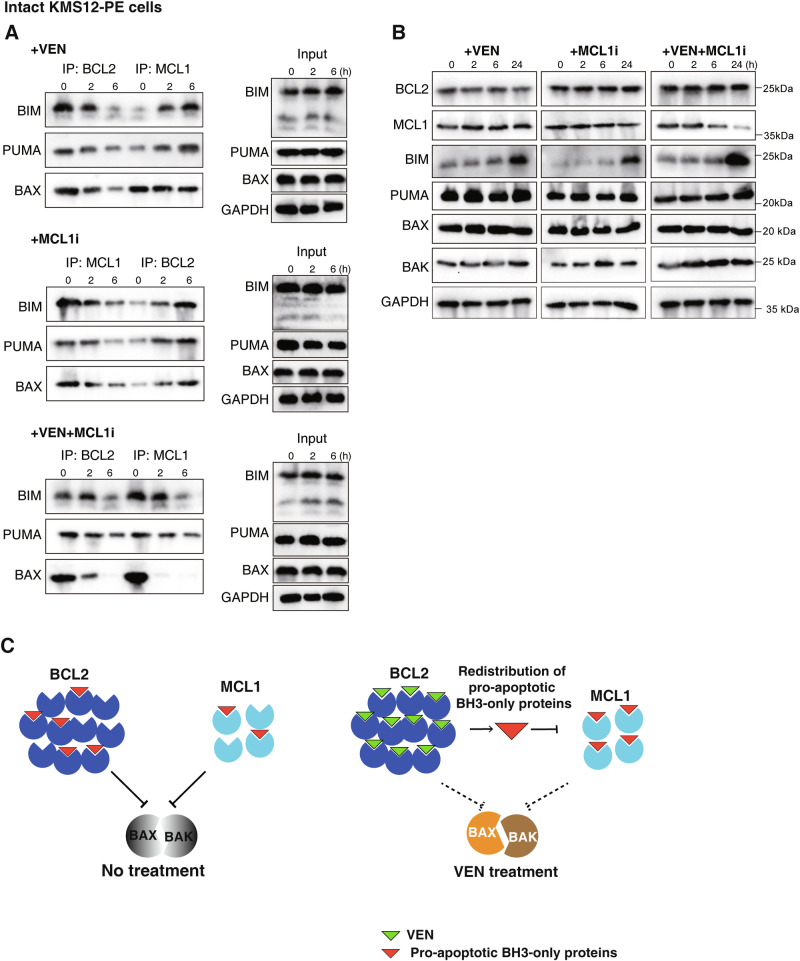
Impact on the sequestration of pro-apoptotic proteins by BCL2 or MCL1 after BH3 mimetic treatment. **A** Redistribution of pro-apoptotic proteins following BH3 mimetic treatment. The amount of BIM, PUMA or BAX bound to BCL2 or to MCL1 in KMS-12-PE after treatment with 1 µM VEN, MCL1i or both inhibitors were examined by co-immunoprecipitation; the cells were co-treated with the broad-spectrum caspase inhibitor Q-VD-OPh (10 µM). Original uncropped immunoblots are provided with Supplemental materials. **B** Levels of BCL2 family proteins after treatment with BH3 mimetics. Lysates prepared from KMS-12-PE cells after treatment with 1 µM VEN, MCL1i or both for 0–24 h were blotted for the indicated proteins; as in (**A**), the experiments were performed with the addition of Q-VD-OPh. Original uncropped immunoblots are provided with Supplementary materials. **C** Working model for the indirect impact on MCL1 triggered by targeting BCL2 with venetoclax in CLL cells. The capacity of BCL2/MCL1 to sequester BAX/BAK in CLL cells is influenced by the abundance of prevailing endogenous BH3-only proteins (e.g., BIM, PUMA). Targeting BCL2 *directly* with venetoclax could *indirectly* inhibit MCL1 (and other pro-survival proteins) because of the displacement of BH3-only proteins from BCL2 caused by venetoclax [49] or redistribution of newly synthesized new proteins Co-targeting BCL2 and MCL1 frees the pro-apoptotic proteins from control by both pro-survival protein maximizing cell killing. Blots in (**A**) and (**B**) are representatives of 2 independent experiments; blotting for GAPDH served as loading controls.

Finally, we sought biochemical evidence for direct and indirect consequences of pharmacologically targeting BCL2 or MCL1 using KMS-12-PE cells as a model system. As expected, inhibiting BCL2 led to the reduction of bound BH3-only proteins (BIM, PUMA) and the cell death mediator BAX (Fig. 7A; upper panels) consistent with direct inhibition of BCL2 and consequent displacement of these pro-apoptotic proteins. Concomitantly, there were increased amounts of these pro-apoptotic molecules bound to MCL1 (indirect effect). MCL1i treatment had the opposite effect (Fig. 7A; middle panels) whereas targeting both BCL2 and MCL1 led to displacement of the pro-apoptotic molecules from both pro-survival proteins (Fig. 7A; lower panels). Importantly, these changes occurred soon (by 6 h) after intact cells were treated as any changes in protein expression (e.g. increased BIM; Fig. 7B) only occurred late.

Taken together, our data studying KMS-12-PE cells support a model whereby targeting the main pro-survival factor e.g. BCL2 with venetoclax has indirect consequences on the ancillary survival factor (MCL1).

## Discussion

Our unexpected finding that permeabilized CLL cells are as sensitive to the induction of MOMP by drugs inhibiting MCL1 or BCLxL as to venetoclax (Fig. 1A) led us to investigate why assaying the inhibitors of the pro-survival BCL2 proteins with BH3 profiling do not reliably reflect the activity of such drugs on intact cells. In large measure, this is because most cells express multiple pro-survival BCL2 proteins and, critically, all the barriers to BAX/BAK-driven MOMP must be removed to enable mitochondrial depolarization to proceed in the permeabilized cells treated with an inhibitor (Figs. 3B, 5A, C, E).

Unlike conventional cell viability assays whereby intact cells are exposed to drugs for hours to several days, the assays for mitochondrial depolarization utilizing permeabilized cells are undertaken within shorter incubation times (<2 h). Longer incubations with permeabilized cells in the laboratory are generally unfavorable as mitochondria rapidly lose their integrity over time [16, 47, 48]. Since drug exposure times are likely to be critical for assessing the potency of a BH3 mimetic, the longer exposure of intact cells to an inhibitor could allow the drug to eventually overcome any block imposed by ancillary pro-survival proteins. Accordingly, just as the efficacy of MCL1i on permeabilized AMO1 cells for 1.5 h was greatly enhanced by removing *BCL2*, the killing of intact AMO1 cells by MCL1i was also increased by co-targeting BCL2 especially at the earlier time points. Co-targeting BCL2 contributed less at later time-points (Fig. 3G, H).

When a BH3 mimetic specifically and rapidly inhibits a key pro-survival protein within a cell (e.g., AMO1 treated with MCL1i), ancillary non-targeted pro-survival proteins (BCL2 in AMO1; Fig. 3G, H) must also be eventually neutralized. With CLL, venetoclax treatment must ultimately also inhibit MCL1 (Fig. 6D). Mechanistically, this is likely to be mediated by the displacement of pre-bound BH3-only proteins (Fig. 7B) as reported in CLL [49] or, potentially, by the redistribution of pro-apoptotic proteins that are newly-made. Both would lead to secondary inhibition of the ancillary pro-survival proteins e.g., MCL1 in CLL (see proposed working model in Fig. 7C).

Moreover, permeabilized cells present a biophysical environment markedly different from that in intact cells which could compromise the capacity of BH3 mimetics to have secondary impacts on ancillary survival proteins that they do not target directly. For instance, the local concentrations or compartmentalization of protein factors are altered after permeabilization, and new proteins are no longer synthesized. Such changes, and others, might also explain the failure of assays with permeabilized cells to faithfully recapitulate the behavior of inhibitors on intact cells.

Taken together, our studies (dose titrations, validating the specificity of the reagents used) revealed that results from BH3 profiling need to be judiciously interpreted and require validation by orthogonal methods. Nevertheless, our work illustrates the requirement to fully overcome both the dominant and the ancillary pro-survival barriers preventing MOMP and identifies factor(s) that limit BH3 mimetic action in diverse hematological malignancies (Fig. 6, S5). Deeper therapeutic responses should be expected to result from co-targeting ancillary survival factors and will likely lead to improved therapeutic outcomes by reducing or eliminating resistant disease, even in complete clinical responders. Recent observations [50–53] that increased MCL1 expression is a prominent driver of clinical resistance to venetoclax in CLL gives further impetus to developing such a strategy.

## Materials and methods

### Patient samples

Peripheral blood or bone marrow samples were collected from patients after written informed consent was obtained. Studies were performed with the approval of the institutional Human Research Ethics Committees at WEHI (approval 05/04), the Royal Melbourne Hospital, the Monash Medical Centre, or the Alfred Hospital. The patient information is summarized in Table S1.

### Mice

Female 6–8-week-old immunodeficient NOD SCID IL-2Rγ^-/-^ (NSG) mice were used for in vivo tumor growth studies. All animal experimentation was approved by the Walter and Eliza Hall Institute Animal Ethics Committee (AEC approval 2017.007) and conducted according to its guidelines.

### Cell lines

All cancer cell lines were cultured in RPMI-1640 medium supplemented with 10% fetal bovine serum (FBS) with the following exceptions: AMO1 cells were cultured in RPMI-1640 medium supplemented with 20% FBS; H929 cells in RPMI-1640 medium supplemented with 10% FBS and 50 µM 2-mercaptoethanol. All cell lines were cultured at 37 °C with 5% CO_2_. The human embryonic kidney cell line HEK293T was cultured in DMEM supplemented with 10% FBS at 37 °C with 10% CO_2_. All media contained 1% penicillin and streptomycin (Sigma #P4333). Regular authentication of all cell lines was performed using STR (short tandem repeat) profiling at Cell Bank Australia. Cells were tested monthly using the MycoAlert Mycoplasma Detection Kit (Lonza #LT07) and were consistently negative for mycoplasma. All the gene knockout cell lines were generated using CRISPR/Cas9 gene editing using published protocols and reagents [54, 55].

### Plasmids

Constitutive Cas9 vector FUCas9Cherry (Addgene #70182) and inducible guide RNA vector FgH1tUTG (Addgene #70183) have been described [55]. pLV hUbC-dCas9 VP64-T2A-GFP (Addgene #53192), phU6-gRNA (Addgene #53188), pmU6-gRNA (Addgene #53187), phH1-gRNA (Addgene #53186) and ph7SK-gRNA (Addgene #53189) for multiplex CRISPR/Cas9-based genome engineering have been described [54]. Details of the guide RNAs and sequencing primers are provided in Table S2.

### Recombinant peptides

BH3-only peptides were synthesized by GenScript, and sequences of the peptides used are provided in Table S2.

### Chemicals

The following compounds were sourced commercially—venetoclax (Active Biochem #A-1231), ABT-737 (Active Biochem #A-1002) and MCL1i (S63845, Active Biochem #A-6044). BCLXLi (A-1331852) was synthesized at WEHI by P Lan and G Lessene. Q-VD-OPh was purchased from MedChemExpress (MCE).

### CRISPR/Cas9 gene editing

Cells were serially infected with lentiviruses expressing Cas9 mCherry and sgRNA GFP/CFP. To induce expression of the sgRNA, doxycycline (Sigma #D9891) was added to the tissue culture medium at a final concentration of 1 μg/mL. Deletion of the targeted protein in the pool of engineered cells expressing sgRNAs was confirmed by immunoblotting. To generate knock-out cell lines, single cells were sorted into individual wells of a 96-well plate. Insertions or deletions (indels) were confirmed by sequencing using the Illumina MiSeq platform [55]. Cells harboring quadruple knock-outs of *BCL2*, *BCLXL*, *BCLw* and *BCL2-A1* in AMO1 cells were generated using multiplex CRISPR/Cas9-based genome engineering as described [54].

### Virus production and cell infection

Lentivirus packaging plasmids (pMDLg/pRRE, pRSV-Rev, pCMV VSV-G) or retrovirus packaging plasmids (gag/pol, pCMV VSV-G) were transiently transfected into HEK293T cells with the constructs of interest using FuGENE 6 Transfection Reagent (Promega #E2691). Supernatants containing infectious virus particles were harvested 48 h later. A second viral harvest was performed following a further 24 h incubation with fresh medium. Supernatant containing virus was filtered through a 0.45 µm filter and stored at 4 °C or –80 °C prior to use. Typically, cells were seeded into 6-well plates at 10^6^ cells/well. An equivalent volume of culture medium containing virus was added along with polybrene (Sigma #H9268) at a final concentration of 5 μg/mL. Cells were spin infected (1800 × *g*, 25 °C, 1 h) and then incubated at 37 °C for 24 h. Cells were then washed and re-suspended in fresh medium.

### Cell viability assays

In total, 5 × 10^3^ cells/well were seeded for CellTiter-Glo assays (Promega #G9243) performed according to the manufacturer’s instructions. Cell viability (%) was calculated by normalizing to the viability of cells treated with DMSO (vehicle control). GraphPad Prism software was used to calculate the concentration at which cell viability was reduced to 50% (IC_50_).

For the caspase activation assays, 5 × 10^3^ cells were seeded per well into 96-well plates and treated with the indicated drugs for up to 6 h. After incubation, 100 μL Caspase Glo reagent (Promega #G8091) was added to each well, and the resulting luminescence was read on a luminometer (LumiStar Optima, BMG Labtech) after 30 min incubation at room temperature.

### BLISS scores

The predicted additive effect was calculated using the BLISS model of fractional independence and subtracted from the actual measured combinatorial effect to generate the BLISS scores of combining two compounds (Bliss, 1939 #86): synergistic ( >0) or additive ( <0); an example is provided in Table S3.

### Primary patient samples and drug treatment

Mononuclear cells from patients’ bone marrow or peripheral blood samples were isolated using Ficoll-Paque Plus (Sigma #GE17-1440-02). Buffy layers containing the mononuclear cells were collected and red blood cells were removed using red blood cell lysis buffer (10 mM KHCO_3_, 150 mM NH_4_Cl, 0.1 mM EDTA, pH 8.0) at 37 °C for 5-10 min, then washed with sterile phosphate-buffered saline (PBS).

Ficoll-Paque-purified myeloma cells (data shown in Fig. 5A, S5B) were resuspended in RPMI-1640 medium supplemented with 20% FBS and 1% penicillin/streptomycin. Equal numbers of cells were then treated with the BH3 mimetic either alone (100 nM) or in equimolar combinations (1:1 or 1:1:1 ratios) for 24 h. Cells were then collected and stained with PE-Cy7-CD38 (BD Biosciences #560677), V450-CD45 (BD Biosciences #560367), FITC-Annexin V (BD Biosciences #560931) and PI (Sigma #P4864) for 45 min. PE-Calibrite beads (BD Biosciences #P340486) were added to each sample before FACS analysis. The number of live myeloma cells (CD38^+ve^/CD45^-ve^/PI^-^ve/Annexin^-ve^) was then determined using an LSR-Fortessa flow cytometer (BD Biosciences) and the percentage cell viability was calculated by normalizing to the sample treated with DMSO. Alternatively, primary samples derived from relapsed/refractory myeloma patients were treated with venetoclax (500 nM), MCL1i (100 nM), or combining both agents. Viable myeloma cells (CD38^+ve^/CD45^-ve^) were analyzed 72 h later by Apo2.7 (Beckman Coulter #IM2088U) staining and cell viability was normalized to that with no drug treatment.

Ficoll-Paque-purified AML cells were resuspended in RPMI-1640 medium supplemented with 15% FBS and 1% penicillin/streptomycin and seeded in 6-well plates at 2.5 x 10^5^ cells/well. Cells were then treated with serially-diluted concentrations (0–10 μM, 5-point, 1:10 dilutions) of the BH3 mimetics either alone or in equimolar combinations (1:1 or 1:1:1 ratio) for 48 h. Cell viability was determined by SYTOX Blue Dead Cell Stain (Life Technologies #S34857) exclusion using an LSR-Fortessa flow cytometer.

Ficoll-Paque-purified chronic lymphocytic leukemia (CLL) cells were resuspended in IMDM supplemented with 10% FBS and 1% penicillin/streptomycin and seeded in 96-well plates at 1 × 10^5^ cells/well. Cells were treated with BH3 mimetics individually (0–10 μM, 5-point, 1:8 dilutions) or in a combination matrix that paired every concentration of both drugs. After 1.5–24 h of treatment, the viability of CLL cells (CD5^+ve^/CD19^+ve^) was determined by PI/Annexin V staining using an LSR-Fortessa flow cytometer. The BLISS scores were calculated as described above (see **BLISS score**).

### Mitochondrial depolarization assays (BH3 profiling)

Cells were washed and resuspended in DTEB buffer (135 mM trehalose dehydrate, 10 mM HEPES-KOH, 50 mM KCl, 20 μM EDTA, 5 μM EGTA, 0.1% w/v BSA, 5 mM succinic acid, pH7.5) at a density of 1 × 10^6^ cells/mL; 100 μL cell suspension was then seeded per well in a 96-well plate. To test the mitochondrial response to different compounds, 100 μL DTEB solution containing 0.002% digitonin (Sigma #D141), 20 μg/mL oligomycin A (Sigma #75351) and the test compound (0–10 μM, 9-point, 1:4 dilutions) were added to the cells in a 96-well plate and incubated in the dark at room temperature for 0–2 h. The fluorescent dye JC-1 (Sigma #T4069) was then added (25 μL, final concentration 900 nM) and incubated for another 45 min followed by flow cytometric analysis. The retention of JC-1 in mitochondria was measured from the 488 nm argon-ion laser using a 530/30 nm filter (FITC, green fluorescence) and a 585/15 nm filter (PE, red fluorescence) on an LSR-Fortessa flow cytometer. The depolarization of mitochondria was indicated by a decrease in the red/green fluorescence intensity ratio. Cells treated with DMSO and FCCP (Sigma #C2920) served as the negative and positive controls respectively.

For CLL samples, Ficoll-Paque-purified mononuclear cells were stained with APC-anti-CD5 (Beckman Coulter #B55386) and BV510-anti-CD19 (BD Biosciences #562947) antibodies at 4 °C for 30 min in FACS buffer (KDS.BSS buffer supplemented with 2% FBS and 0.02% sodium azide) followed by assaying for mitochondrial depolarization after permeabilization.

### Immunoblotting

Whole cell lysates were generated using lysis buffer containing 1% Triton X-100 and complete protease inhibitors. Protein content was quantified using the Bradford assay (Bio-Rad #5000002). Lysates were diluted with 4 x SDS reducing buffer and denatured by boiling at 95 °C for 10 min. Proteins (40 μg) were separated by SDS-PAGE using NuPAGE 4–12% Bis-Tris gels (Invitrogen) and transferred onto nitrocellulose membranes (Invitrogen #LC2009) using the iBlot2 Dry Blotting System (Thermo Fisher Scientific). Membranes were blocked at room temperature (RT) for 1 h in blocking buffer (PBS containing 0.1% Tween-20 (PBST) and 5% (w/v) skimmed milk powder). Membranes were then incubated overnight at 4°C in primary antibody diluted in blocking buffer. The next day, membranes were washed in PBST and incubated for 1 h at RT in HRP-conjugated secondary antibodies diluted in blocking buffer. Membranes were washed again in PBST and imaged by the addition of Immobilon Forte (Merck Millipore #WBLUF0500) on a ChemiDoc system (Bio-Rad). Monoclonal antibodies to BCL2 (Abcam #ab182858), MCL1 (Abcam #ab243136), BAX (CST #5023S), BAK (CST #12105S), BIM (CST #2933S), PUMA (CST #98672S), Cytochrome *c* (BD Biosciences #556433), β-actin (Sigma #A4700) and GAPDH (OriGene # TA-08) were used in this study; all antibodies were diluted in blocking buffer.

### Co-immunoprecipitation

In total, 4 × 10^7^ cells per sample were lysed in 500 μL lysis buffer. Pre-cleared lysates were then incubated with the antibodies at 4 °C for 3 h followed by incubation with Protein A/G agarose beads (Beyotime, #P2029) at 4 °C overnight. Beads were then collected by centrifugation at 3000 rpm at 4 °C for 1 min and washed with PBS for 10 times. Bound proteins were eluted with 50 μL 2 × SDS reducing buffer and denatured by boiling at 95 °C for 10 min. Immunoprecipitation (IP) and input (input) samples were analyzed by immunoblotting.

### Cytochrome c release assay

In total, 3 × 10^6^ cells were washed, resuspended in 100 μL DTEB buffer containing 0.01% digitonin and titrated concentrations of BH3 mimetic compounds, followed by incubation in the dark at room temperature for 1 h. Samples were then spun at 13,000 rpm at 4 °C for 5 min to separate the soluble from the pellet (mitochondria-containing) fractions. The latter was then lysed by adding 100 μL lysis buffer (20 mM Tris-HCl pH 7.4, 135 mM NaCl, 1.5 mM MgCl_2_, 1 mM EDTA, 10% glycerol) containing 1% Triton X-100 and complete protease inhibitors (Roche #4693132001). Both fractions were then diluted with 4 x SDS reducing buffer and denatured by boiling at 95 °C for 10 min. The amounts of Cytochrome *c* and β-actin in the fractions were determined by immunoblotting.

### In vivo studies

In total, 1 × 10^7^ wild-type or *BCL2*^–/–^ AMO1 cells expressing luciferase were injected intravenously into 6–8-week-old female NSG mice. Mice underwent baseline imaging on day 10 using the in vivo imaging system (IVIS, Perkin Elmer) and were assigned into 2 groups: control group (treated with vehicle), or with 25 mg/kg MCL1i on days 11, 14, 18 and 21. Mice were euthanized when deemed unwell by experienced animal technicians.

In total, 1 × 10^7^ wild-type AMO1 cells expressing luciferase were injected intravenously into 6–8-week-old female NSG mice. Mice underwent baseline imaging on day 10 using the in vivo imaging system (IVIS, Perkin Elmer) and were assigned into 4 groups: control group (untreated), 50 mg/kg venetoclax on days 10–14 and days 17–21, 25 mg/kg MCL1i on days 11, 14, 18 and 21, or with both compounds. On day 27, the whole-body tumor burden was determined by imaging, mice were then euthanized and the level of CD38^+^ (BD Biosciences #560677) tumor cells in the peripheral blood and bone morrow was determined by flow cytometry. Mice were euthanized when deemed unwell by experienced animal technicians.

In total, 1 × 10^7^ KMS-12-PE cells were inoculated subcutaneously into the right flank of 6–8-week-old female NSG mice. The inoculation volume (100 μL per mouse) contained a 50:50 mixture of cells in PBS and Matrigel (Corning #354248). When tumors became palpable after 10 days, mice were randomized into 5 groups: control (no treatment) group, 25 or 100 mg/kg venetoclax from days 10–14 and days 17–21, 25 mg/kg MCL1i on day 11, 14, 18 and 21, or with 25 mg/kg of both drugs. Venetoclax was formulated for oral dosing in 60% phosal 50 propylene glycol (Lipoid #FSHNC0130871), 30% polyethylene glycol (PEG)-400 (Sigma #202398) and 10% ethanol. MCL1i was formulated for i.v. injection in 50% 2-hydroxypropyl-β-cyclodextrin (Sigma-Aldrich #H107) and 50% 50 mM HCl. An electronic caliper was used to measure the length and width of tumors every 2–3 days. Tumor volume was estimated using the following equation: volume = ½ x length x width x width.

### Quantification and statistical analysis

GraphPad Software was used for statistical analysis. All data were expressed as means ± standard deviation (SD). Statistical significance was analyzed using unpaired Student’s *t* test, with *p* values < 0.05 considered to be statistically significant.

## Supplementary information

Supplemental material

Gong et al original western blots

## Data Availability

The original contributions presented in the study are included in the article or the supplementary material. This paper does not report original code. Other data that support the findings are available from the corresponding author upon reasonable request
