# Supplementary material for: Re-appraising assays on permeabilized blood cancer cells testing venetoclax or other BH3 mimetic agents selectively targeting pro-survival BCL2 proteins
Source: Cell Death Differ. 2025 Apr 9;32(8):1382–96. doi: 10.1038/s41418-025-01487-7 (PMC12325916; doi:10.1038/s41418-025-01487-7)
Supplement: Supplementary file 1 — Supplemental material [file 41418_2025_1487_MOESM1_ESM.pdf]

Supplementary information

Table S1 Patient information

Table S2 Details of guide RNAs, sequencing primers and peptides used

Table S3 BLISS score calculation

Supplementary figures and legends

Table S1

Patients with multiple myeloma

	Gender	Age	Status at analysis	Previous Rx	Cytogenetics	WCC Count (X10^9/L)	PP (g/L)	Isotype
MM #1	M	61	New	Nil	Normal Karyotype, (FISH negative for del17p and t(4;14))	3.5	24	IgG Lambda
MM #2	M	42	New	Nil	Normal Karyotype, 46,XY(51].nuc ish(FGFR3,IGH)x2[100),(TP53,NF1)x3[51/100]	4.3	Sample was collected on 20181130, 10 g/L on 20171124	IgA Kappa; Kappa FLC 400 mg/L; Lambda FLC less than 4.5 mg/L on 20171124
MM #3	M	48	New	Nil	N/A	2.7	Sample was collected on 20171220, 3 g/L on 20180426	IgG Kappa; Kappa FLC 31.9 mg/L; Lambda FLC 7.6 mg/L
MM #4	M	62	New	Nil	N/A	5.8	10	IgG Kappa; Kappa FLC 17.8 mg/L; Lambda FLC 8.9 mg/L
MM #5	M	62	New	Nil	N/A	6.1	20	IgM Kappa & IgM lambda comigrating & totalling 20g/L (monoclonal IgM Kappa)
MM #6	M	73	New	Nil	Normal Karyotype, (FISH negative for del17p and t(4;14))	3.7	31	IgG Lambda; Kappa FLC 4.0 mg/L; Lambda FLC 124 mg/L
MM #7	M	81	New	Nil	Normal Karyotype (#Y), (FISH negative for del17p and t(4;14))	3.3	41	IgG Kappa
MM #8	F	56	New	Nil	Hyperdiploid 53, XY, +3, +5, +6, +7, +9, +15, +19, add (19) (q13), +mar[3]/53, idem, add(5)(q31), add(11) (q13), #add(19), #mar[4]/46, XY[15]. nuc ish (FGFR3X2, IGHX3) [35/100], (CCND1X2, IGHX3) [42/100], (TP53, NF1) x2[100]	9.7	Sample was collected on 20180215, 9 g/L on 20180221	IgD Lambda; Kappa FLC 7.3 mg/L; Lambda FLC 1412 mg/L on 21/02/18
MM #9	M	50	New	Nil	N/A	18	Sample was collected on 20180216, not detected on 21/02/18	Normal levels of polyclonal gamma globulins were detected. The electrophoretic pattern was essentially normal; Kappa FLC less than 0.50 mg/L; Lambda FLC 32.9 mg/L on 21/2/18
MM #10	M	45	New	Nil	N/A	8.1x109/L	Sample was collected on 20180627, 2 g/L IgA on 20180614	Monoclonal IgA Lambda Kappa ligt chain (16.5 mg/L) Lambda ligt chain (12.4 mg/L)
MM #11	F	71	RR	Thalidomide	Normal Karyotype, 46, XX (25)	6.7	N/A	Kappa Light chain
MM #12	F	65	New	Nil	Normal Karyotype	4.2 x109/L	Sample was collected on 20180705, 14 g/L IgA on 20180619	Monoclonal IgA Lambda Kappa ligt chain (9.0 mg/L) Lambda ligt chain (11.4 mg/L)
MM #13	F	59	RR	N/A	N/A	N/A	N/A	N/A
MM #14	M	57	RR	N/A	Non-Hyperdiploid, del 13, 14, 18, 22. 12p+, 1q+, t(4;14)	N/A	N/A	N/A
MM #15	M	62	RR	N/A	Hyperdiploid, 1q+, 17p-, 13q-	N/A	N/A	N/A
MM #16	M	68	RR	N/A	N/A	N/A	N/A	N/A
MM #17	M	70	RR	N/A	N/A	N/A	N/A	N/A
MM #18	M	68	RR	N/A	N/A	N/A	N/A	N/A
MM #19	M	43	RR	N/A	N/A	N/A	N/A	N/A
MM #20	F	67	RR	N/A	N/A	N/A	N/A	N/A
MM #21	M	53	RR	N/A	Non-Hyperdiploid, t(11;14), 1(1;16), del 1, 14, 16	N/A	N/A	N/A
MM #22	M	51	RR	N/A	17p-	N/A	N/A	N/A

ND = newly diagnosed. RR = relapsed/refractory. PP = paraprotein level
HRD = hyperdiploid
N/A: not available

Table S1-continue

Patients with acute myeloid leukemia (AML)

Primary AML	Primary AML	Age	Gender	Status at analysis	WHO2008	WCC (x10^9/L)	Blasts (%)	Starting viability	Karyotype	MRC2010Risk
03-331-2018	AML_1	21	M	New	N/A	3.39	83.6	86.2	Pseudohypodiploid, unbalanced 2;5, unidentified	Poor
04-341-2018	AML_2	38	F	New	N/A	19.9	78	78.8	Normal	Intermediate
02-190-2018	AML_3	61	F	New	N/A	46.05	80.4	69.6	Normal	Intermediate
02-194-2018	AML_4	68	F	New	N/A	224.96	82.6	67	Normal	Intermediate
02-232-2018	AML_5	27	M	New	AML with mutated CEBPA	66.49	88.6	78.9	Normal	Intermediate
04-015-2018	AML_6	51	M	New	AML with mutated NPM1	46.89	98	77.4	Normal	Intermediate
BXH008	AML_7				N/A			77.9		
01-072-2018	AML_8	57	M	New	AML with mutated NPM1	39.87	86.6	94.1	Normal	Intermediate
03-330-2018	AML_9	65	F	New	N/A	3.12	65	76.1	Normal	Intermediate
01-324-2017	AML_10	33	M	New	Mixed phenotype acute leukaemia, B/myeloid NOS	9.97	81	77.7	Normal	Intermediate
03-204-2018	AML_11	66	F	New	N/A	43.13	41	74.4	t(3;3)	Poor
01-218-2018	AML_12	47	F	New	N/A	2.86	79.2	97	Trisomy 11	Intermediate

Table S1-continue

Patients with chronic lymphocytic leukemia (CLL)

Primary CLL	Age	Gender	Status at analysis	Previous Rx	WCC	Lymphocytes (%)	$CD5^{+}CD19^{+}$cells (%)	Karyotype	Del17p	TP53 mut	IgVH
CLL#1	54	M	Unknown	N/A	60	90	86	N/A	N/A	N/A	N/A
CLL#2	62	F	Unknown	N/A	21	68	53	N/A	N/A	Not detected	Mutated
CLL#3	63	M	RR	chlorambucil, rituximab	20	75	86	N/A	Not detected	5-10% CLL cells have Arg248Gln missense mutation	N/A
CLL#4	79	M	De novo	nil	50	84	95	Complex	N/A	Not detected	N/A
CLL#5	55	M	De novo	nil	149	80	88	Normal	Not detected	Not detected	N/A
CLL#6	71	F	RR	Chlorambucil, Navitoclax	20	93	82	Normal	Not detected	Not deteced	Mutated

Table S2

Oligonucleotides	
**guide RNAs**	
h*BCL2* sgRNA: GCGGCGGGAGAAGTCGTCGC	Gong et al, Blood, 2016
h*BCLXL* sgRNA: GGCCTTTTTCTCCTTCGGCG	Gong et al, Blood, 2016
h*BCLw* sgRNA: GGAGTTCACAGCTCTATACG	Gong et al, Blood, 2016
h*BCL2A1* sgRNA: GTCCTACAGATACCACAACC	Gong et al, Blood, 2016
h*BAX* sgRNA #1: CTGCAGGATGATTGCCGCCG	Gong et al, Blood, 2016
h*BAK* sgRNA #1: GCATGAAGTCGACCACGAAG	Gong et al, Blood, 2016
**Sequencing primers**	
h*BCL2* sgRNA sequencing primer-F: GTGACCTATGAACTCAGGAGTCCTCAGCCCGGTGCCACCT	Gong et al, Blood, 2016
h*BCL2* sgRNA sequencing primer-R: CTGAGACTTGCACATCGCAGCTCCCTGAAGAGCTCCTCC	Gong et al, Blood, 2016
h*BCLXL* sgRNA sequencing primer-F: GTGACCTATGAACTCAGGAGTCGGGCATTCAGTGACCTGACA	Gong et al, Blood, 2016
h*BCLXL* sgRNA sequencing primer-R: CTGAGACTTGCACATCGCAGCCACACAAGGGGCTTGGTTCT	Gong et al, Blood, 2016
h*BCLw* sgRNA sequencing primer-F: GTGACCTATGAACTCAGGAGTCCGCAGTGGATGGAACTGGAA	Gong et al, Blood, 2016
h*BCLw* sgRNA sequencing primer-R: CTGAGACTTGCACATCGCAGCGCCCTGGACTTTCACTTGCT	Gong et al, Blood, 2016
h*BCL2-A1* sgRNA sequencing primer-F: GTGACCTATGAACTCAGGAGTCCAAGGTGAGCCAGCTCAAG	Gong et al, Blood, 2016
h*BCL2-A1* sgRNA sequencing primer-R: CTGAGACTTGCACATCGCAGCTGATGCCGTCTTCAAACTCC	Gong et al, Blood, 2016
h*BAX* sgRNA #1 sequencing primer-F: GTGACCTATGAACTCAGGAGTCCTTTAGTGTGCGGTGGATGC	Gong et al, Blood, 2016
h*BAX* sgRNA #1 sequencing primer-R: CTGAGACTTGCACATCGCAGCCCTTGAGCACCAGTTTGCTG	Gong et al, Blood, 2016
h*BAK* sgRNA #1 sequencing primer-F: GTGACCTATGAACTCAGGAGTCCTATGGGATGCTCTGCCCAC	Gong et al, Blood, 2016
h*BAK* sgRNA #1 sequencing primer-R: CTGAGACTTGCACATCGCAGCGGTCACAGAGAGGCTAGCAG	Gong et al, Blood, 2016
**BH3 peptides**	
$BH3^{BIM4E}$ peptide: DMRPEIWEAQEERREGDEENAYYARR	Chen et al, Mol Cell, 2005
$BH3^{BIM2A}$ peptide: DMRPEIWIAQEARRIGDEANAYYARR	Lee et al, J Cell Biol, 2008
$BH3^{BIMBAD}$ peptide: NLWAAQRYGRELRRMSDEFVDSFKKG	Chen et al, Mol Cell, 2005
$BH3^{MS1}$ peptide: RPEIWMTQGLRRLGDEINAYYAR	Foight et al, ACS Chem Bio, 2014
$BH3^{BIM}$ peptide: DMRPEIWIAQELRRIGDEFNAYYARR	Chen et al, Mol Cell, 2005

Table S3

Example of BLISS calculation

AMO1

% viable cells after treatment (1.5h)

(Raw data from flow cytometry)

			MCL1i (µM)					
		control	0	0.002	0.02	0.156	1.25	10
VEN (µM)	0	0.86	0.86	0.879	0.788	0.408	0.0502	0.0054
	0.002		0.858	0.87	0.792	0.395	0.0255	0.0113
	0.02		0.863	0.861	0.677	0.215	0.0091	0.0093
	0.156		0.825	0.78	0.54	0.0975	0.0036	0.0047
	1.25		0.827	0.772	0.432	0.0428	0.007	0.0053
	10		0.839	0.618	0.165	0.0064	0.0086	0.0046

% killing

(normalized to control)

			MCL1i (µM)					
			0	0.002	0.02	0.156	1.25	1
VEN (µM)	0		0	-0.022093	0.0837209	0.5255814	0.9416279	0.9937209
	0.002		0.0023256	-0.0116279	0.0790698	0.5406977	0.9703488	0.9868605
	0.02		-0.0034884	-0.0011628	0.2127907	0.75	0.9894186	0.989186
	0.156		0.0406977	0.0930233	0.372093	0.8866279	0.995814	0.9945349
	1.25		0.0383721	0.1023256	0.4976744	0.9502326	0.9918605	0.9938372
	10		0.0244186	0.2813953	0.8081395	0.9925581	0.99	0.9946512

The predict killing if two drugs are additivie

			MCL1i (µM)					
			0	0.002	0.02	0.156	1.25	10
VEN (µM)	0		0	-0.022093	0.0837209	0.5255814	0.9416279	0.9937209
	0.002		0.0023256	-0.0197161	0.0858518	0.5266847	0.9417637	0.9937355
	0.02		-0.0034884	-0.0256585	0.0805246	0.5239264	0.9414243	0.993699
	0.156		0.0406977	0.0195038	0.1210114	0.5448891	0.9440035	0.9939765
	1.25		0.0383721	0.0171268	0.1188805	0.5437858	0.9438678	0.9939619
	10		0.0244186	0.0028651	0.1060952	0.537166	0.9430533	0.9938743

BLISS Score: (observed-predicted)x100

			MCL1i (µM)						
			0	0.002	0.02	0.156	1.25	10	
VEN (µM)	0		0	0	0	0	0	0	
	0.002		0	0.8088156	-0.6782044	1.401298	2.8585181	-0.6875068	
	0.02		0	2.4495673	13.226609	22.607355	4.7994321	-0.451298	
	0.156		0	7.351947	25.108167	34.173878	5.1810438	0.055841	
	1.25		0	8.5198756	37.879394	40.644673	4.7992699	-0.0124662	BILISS SUM
	10		0	27.853029	70.204435	45.53921	4.6946728	0.0776906	358

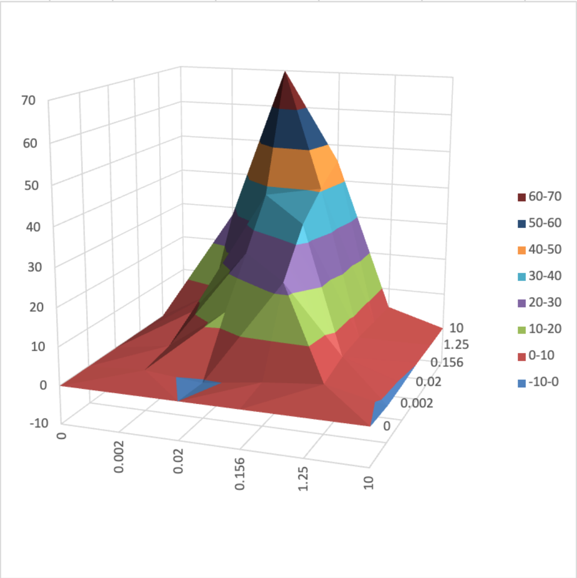

Supplementary figures and legends

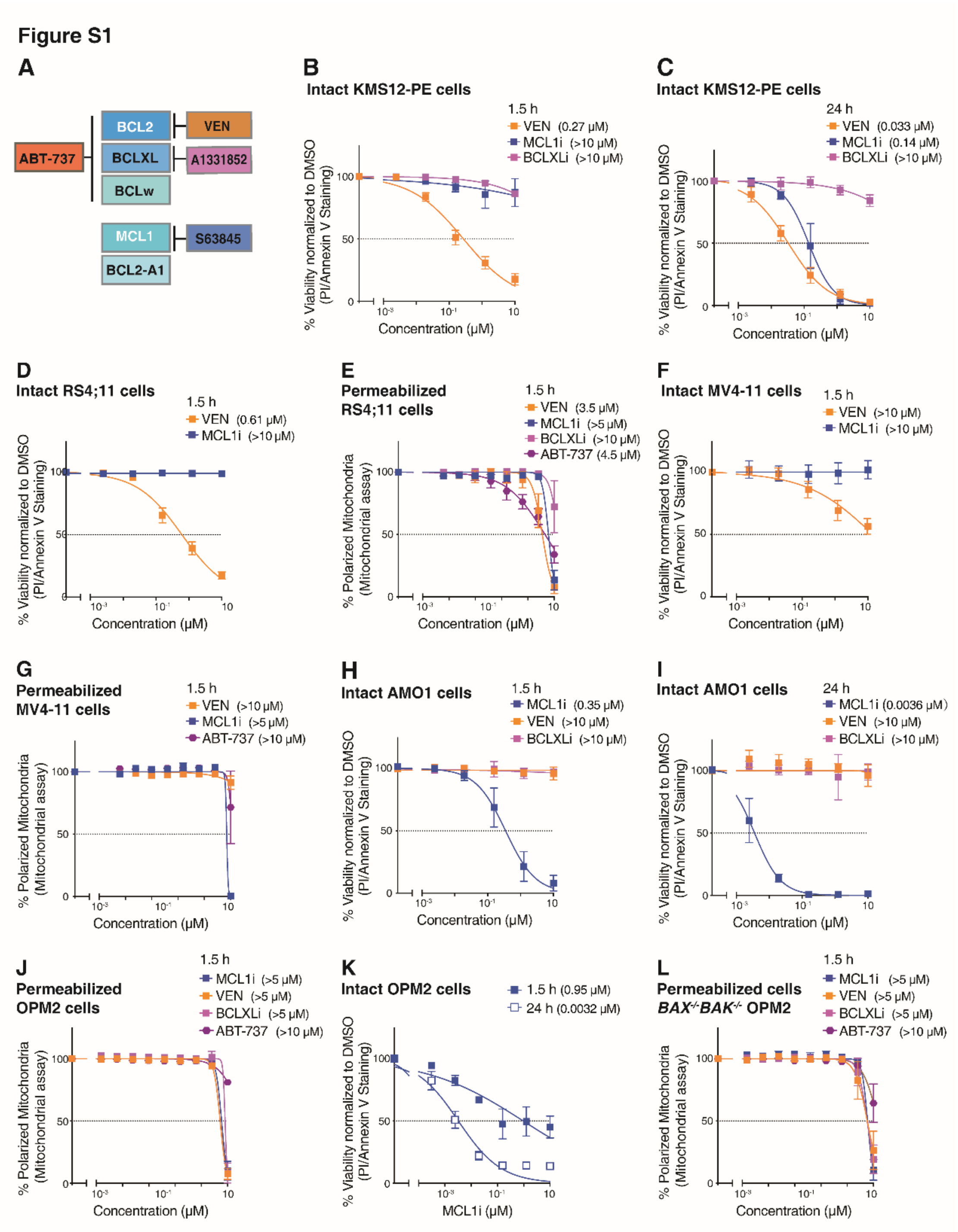

Figure S1 Related to Figure 1

(**A**) Binding specificities of small molecule inhibitors that target the pro-survival BCL2 proteins[1, 2, 3, 4].

(**B**)-(**C**) Response of intact KMS-12-PE cells to the indicated BH3 mimetic at 1.5 hr (**B**) or 24 hr (**C**). Data summarized in Fig. 1D.

(**D**) Response of intact RS4;11 cells to the indicated BH3 mimetic at 1.5 hr.

(**E**) BH3 profiling assays on permeabilized RS4;11 cells treated with the indicated BH3 mimetic at 1.5 hr.

(**F**) Response of intact MV4-11 cells to the indicated BH3 mimetic at 1.5 hr.

(**G**) BH3 profiling assays on permeabilized MV4-11 cells treated with the indicated BH3 mimetic at 1.5 hr.

(**H**)-(**I**) Response of intact AMO1 cells to the indicated BH3 mimetic at 1.5 hr (**H**) or 24 hr (**I**). Data summarized in Fig. 1F.

(**J**) BH3 profiling assays on permeabilized OPM2 cells treated with the indicated BH3 mimetic at 1.5 hr.

(**K**) Response of intact OPM2 cells to the indicated BH3 mimetic at 1.5 hr or 24 hr.

(**L**) BH3 profiling assays on BAX/BAK-deficient OPM2 cells.

Data in (**B**)-(**L**) represent the means ± SD from ≥ 3 independent experiments; IC_{50} are indicated in parentheses.

Figure S2

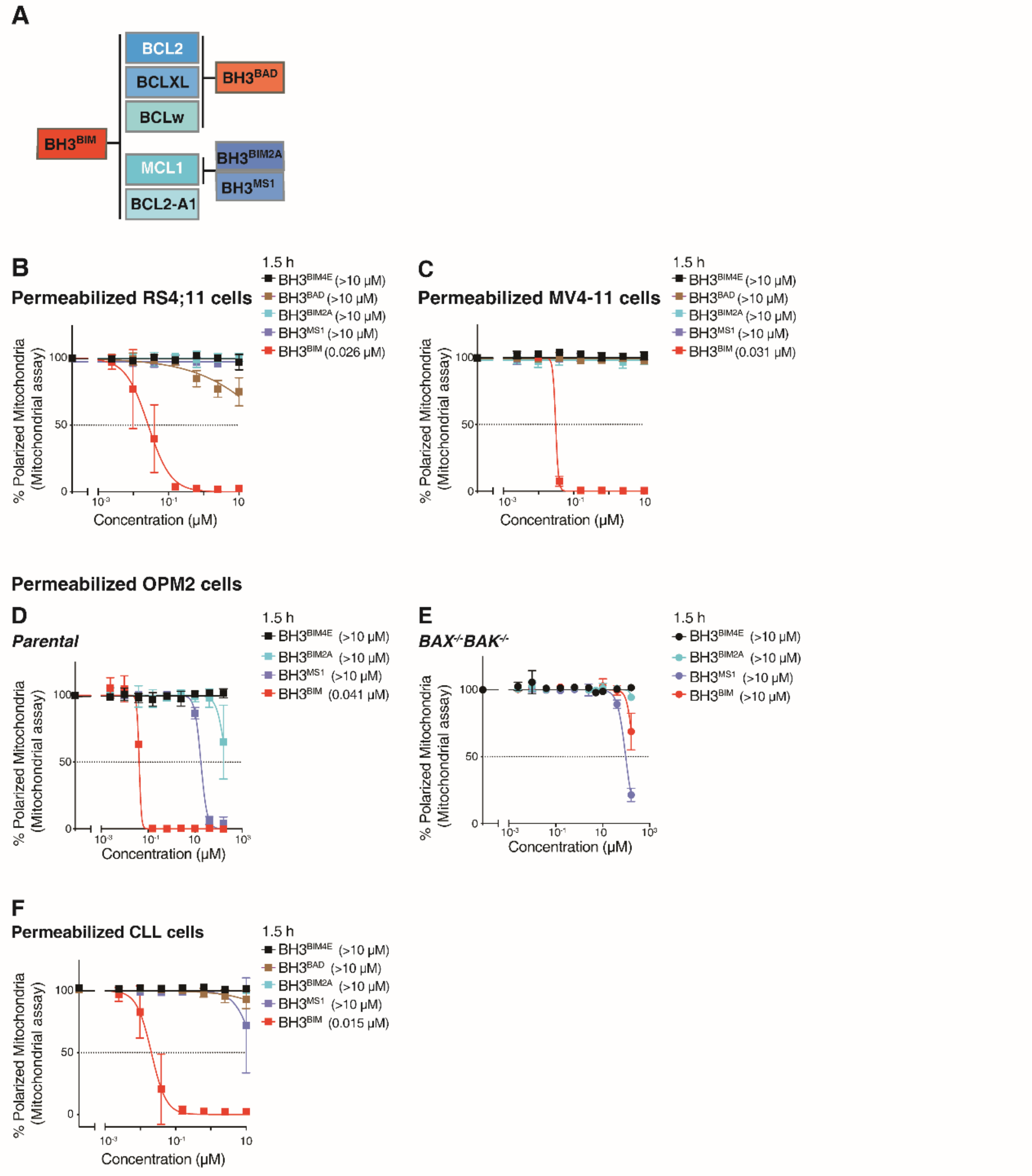

Figure S2 Related to Figure 2

(**A**) Binding specificities of the BH3 peptides for the pro-survival BCL2 proteins[5, 6, 7, 8, 9].

(**B**)-(**C**) BH3 profiling assays on permeabilized RS4;11 (**B**) or MV4-11 cells(**C**).

(**D**)-(**E**) BH3 profiling assays on parental (**D**) or BAX/BAK-deficient OPM2 cells (**E**).

(**F**) BH3^{BIM} peptide induces marked mitochondrial depolarization when added to permeabilized CLL cells. These permeabilized cells were treated with the indicated peptide and mitochondrial depolarization determined 1.5 hr later. Data represent mean

IC50 ± SD of the 6 patient samples studied; the experiment was performed once per sample.

Data in (**B**)-(**E**) represent the means ± SD from ≥ 3 independent experiments. IC50 indicated in parentheses.

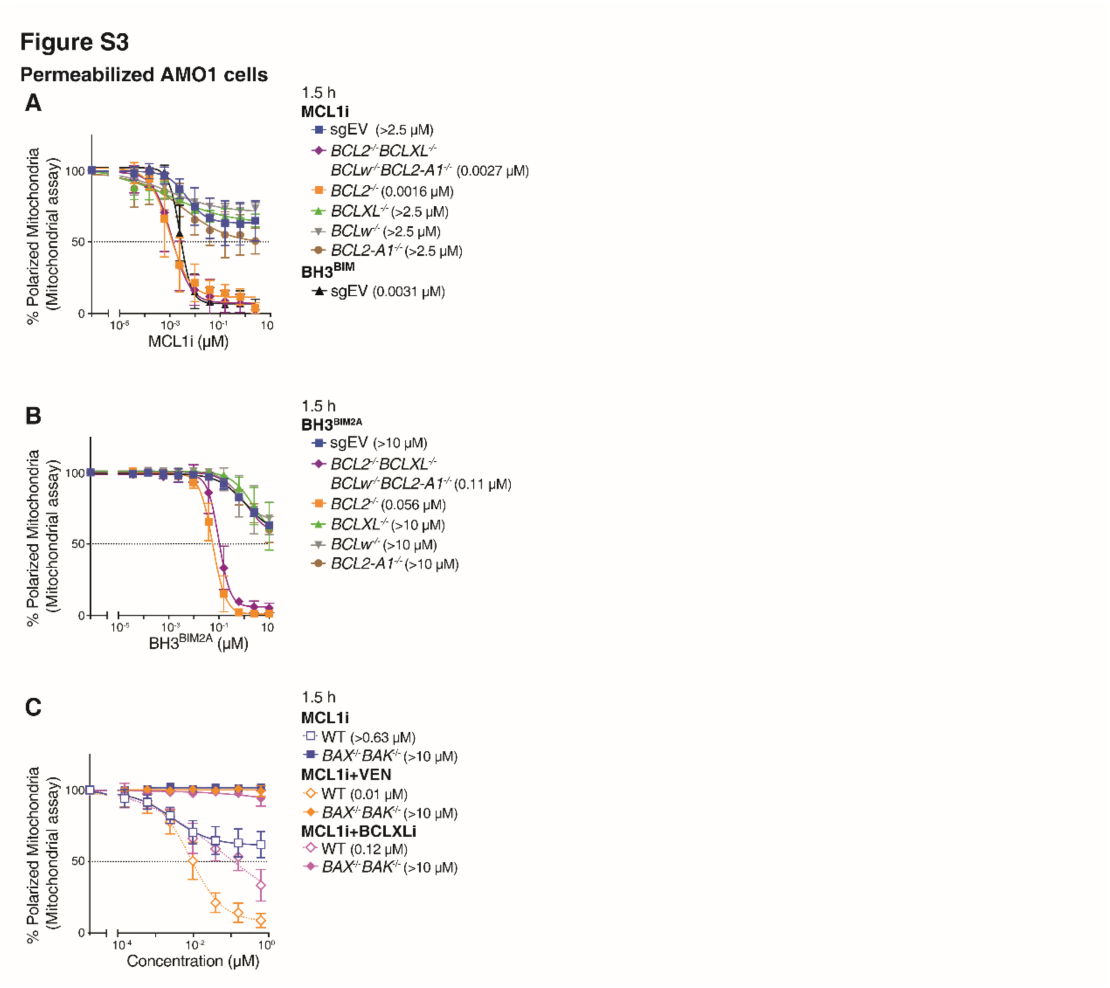

Figure S3 Related to Figure 3

(**A**) BCL2 limits the ability of MCL1i to directly damage AMO1 mitochondria. Representative clones of AMO1 cells of the indicated genotypes were treated with MCL1i for 1.5 hr and mitochondrial integrity was determined. The data (mean IC50s ± SD from 2 independently engineered clones of each genotype) are summarized in Figure 3B.

(**B**) Similar experiments to those in (**A**) were performed with the MCL1-selective $BH3^{BIM2A}$ peptide.

(**C**) BAX/BAK-dependent of killing by BH3 mimetics; full set of the data shown in Figure 3F.

Data represent the means ± SD from ≥ 3 independent experiments. IC_{50} indicated in parentheses.

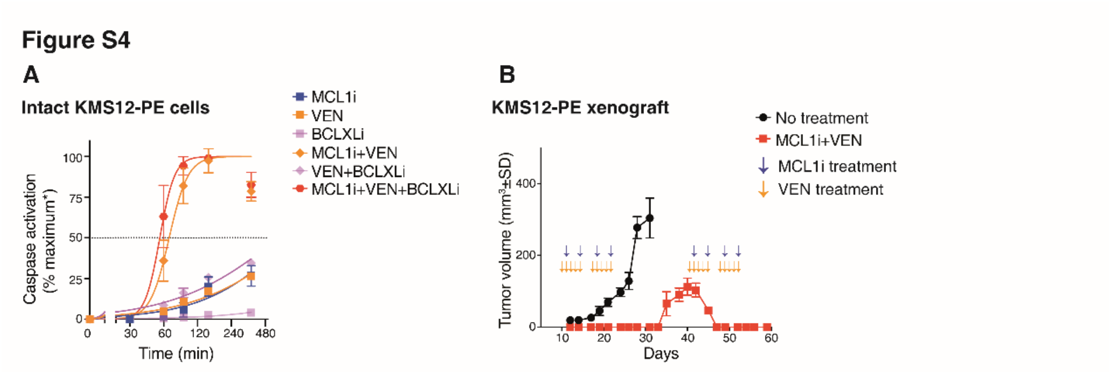

Figure S4 Related to Figure 4

(**A**) Inhibiting MCL1 accelerates venetoclax-induced caspase activation. KMS-12-PE cells were incubated with 100 nM of the indicated BH3 mimetics or equimolar combinations of them (1:1 or 1:1:1 ratios) for the indicated time periods and the activation of caspases was determined by Caspase-Glo 3/7 assay. % caspase activation normalized to the maximum observed with the triple treatment was shown. Data represent the means ± SD of ≥ 3 independent experiments.

(**B**) Addition of an MCL1 inhibitor markedly enhances the activity of venetoclax *in vivo*. Similar experiments to those in Figure 5G were performed, except that mice were given a second round of treatment when tumors recurred. Tumor sizes were measured every 2-3 days and the mean tumor volumes ± SD (3-6 mice per group) are shown.

Figure S5

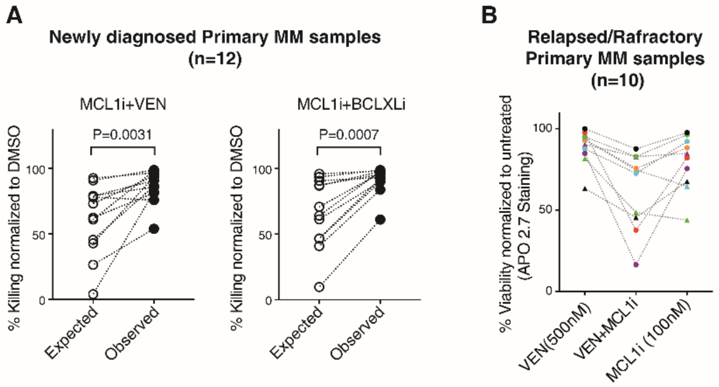

C
Primary AML samples
Intact cells

	IC50s (µM)											
	AML_1	AML_2	AML_3	AML_4	AML_5	AML_6	AML_7	AML_8	AML_9	AML_10	AML_11	AML_12
**VEN**	10	0.86	0.29	10	0.15	0.12	10	10	10	10	10	10
**MCL1i**	1.6	0.8	2.2	0.015	0.2	0.65	3.1	5.5	10	10	10	10
**BCLXLi**	10	10	10	10	10	2.7	10	10	10	10	10	10
**VEN+MCL1i** (7/12，IC50<100nM)	0.082	0.0098	0.036	0.00095	0.0054	0.0017	0.041	0.5	1.6	1.3	6	10
**MCL1i+BCLXLi** (3/12，IC50<100nM)	0.84	0.15	0.87	0.00055	0.06	0.11	0.11	0.16	0.064	8.2	1.3	10
**VEN+MCL1i+BCLXLi** (9/12，IC50<100nM)	0.091	0.008	0.032	0.00043	0.0021	0.0018	0.0066	0.004	0.029	0.13	3.8	10

IC50

Figure S5 Related to Figure 5

(**A**) Synergistic activity of BH3 mimetics. The expected vs. observed effect of MCL1i in combination with venetoclax or BCLXLi in primary myeloma samples was calculated using the Bliss model. Statistical significance was calculated using the paired Student's t-test.

(**B**) Inhibiting both BCL2 and MCL1 showed increased killing in some samples derived from relapsed/refractory myeloma patients. Ten primary samples derived from relapsed/refractory myeloma patients were treated with venetoclax (500 nM), MCL1i (100 nM), or both. Viable myeloma cells ($CD38^{+ve}/CD45^{-ve}$) were analyzed 72 hr later by Apo2.7 staining and normalized to no drug treatment.

(**C**) Table summarizing the response of 12 primary AML samples to BH3 mimetic drugs, given alone or in combination.

Data with these primary samples were generated from one experiment per patient sample. Detailed patient information is provided in Table S1.

References

1. van Delft MF, Wei AH, Mason KD, Vandenberg CJ, Chen L, Czabotar PE*, et al.* The BH3 mimetic ABT-737 targets selective Bcl-2 proteins and efficiently induces apoptosis via Bak/Bax if Mcl-1 is neutralized. *Cancer Cell* 2006, **10**(5)**:** 389-399.

2. Souers AJ, Leverson JD, Boghaert ER, Ackler SL, Catron ND, Chen J*, et al.* ABT-199, a potent and selective BCL-2 inhibitor, achieves antitumor activity while sparing platelets. *Nat Med* 2013, **19**(2)**:** 202-208.

3. Wang L, Doherty GA, Judd AS, Tao ZF, Hansen TM, Frey RR*, et al.* Discovery of A-1331852, a First-in-Class, Potent, and Orally-Bioavailable BCL-X(L) Inhibitor. *ACS Med Chem Lett* 2020, **11**(10)**:** 1829-1836.

4. Kotschy A, Szlavik Z, Murray J, Davidson J, Maragno AL, Le Toumelin-Braizat G*, et al.* The MCL1 inhibitor S63845 is tolerable and effective in diverse cancer models. *Nature* 2016, **538**(7626)**:** 477-482.

5. Letai A, Bassik MC, Walensky LD, Sorcinelli MD, Weiler S, Korsmeyer SJ. Distinct BH3 domains either sensitize or activate mitochondrial apoptosis, serving as prototype cancer therapeutics. *Cancer Cell* 2002, **2**(3)**:** 183-192.

6. Chen L, Willis SN, Wei A, Smith BJ, Fletcher JI, Hinds MG*, et al.* Differential targeting of prosurvival Bcl-2 proteins by their BH3-only ligands allows complementary apoptotic function. *Mol Cell* 2005, **17**(3)**:** 393-403.

7. Kuwana T, Bouchier-Hayes L, Chipuk JE, Bonzon C, Sullivan BA, Green DR*, et al.* BH3 domains of BH3-only proteins differentially regulate Bax-mediated mitochondrial membrane permeabilization both directly and indirectly. *Mol Cell* 2005, **17**(4)**:** 525-535.

8. Lee EF, Czabotar PE, van Delft MF, Michalak EM, Boyle MJ, Willis SN*, et al.* A novel BH3 ligand that selectively targets Mcl-1 reveals that apoptosis can proceed without Mcl-1 degradation. *J Cell Biol* 2008, **180**(2)**:** 341-355.

9. Foight GW, Ryan JA, Gulla SV, Letai A, Keating AE. Designed BH3 peptides with high affinity and specificity for targeting Mcl-1 in cells. *ACS Chem Biol* 2014, **9**(9)**:** 1962-1968.
