# Supplementary material for: Re-appraising assays on permeabilized blood cancer cells testing venetoclax or other BH3 mimetic agents selectively targeting pro-survival BCL2 proteins
Source: Cell Death Differ. 2025 Apr 9;32(8):1382–96. doi: 10.1038/s41418-025-01487-7 (PMC12325916; doi:10.1038/s41418-025-01487-7)
Supplement: Supplementary file 2 — Gong et al original western blots [file 41418_2025_1487_MOESM2_ESM.pdf]

Figure 2E

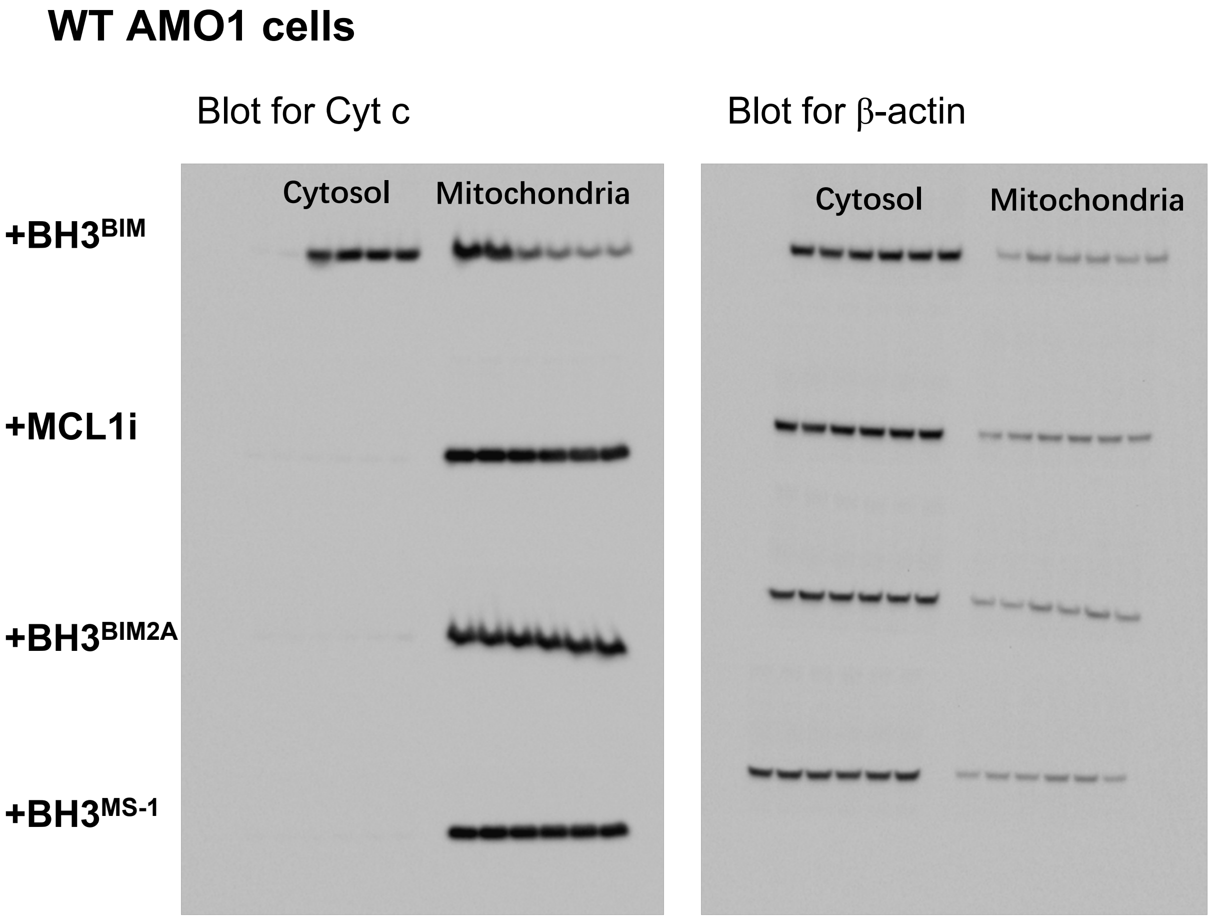
WT AMO1 cells
Blot for Cyt c
Blot for β-actin
Cytosol
Mitochondria
Cytosol
Mitochondria
+BH3^BIM
+MCL1i
+BH3^BIM2A
+BH3^MS-1

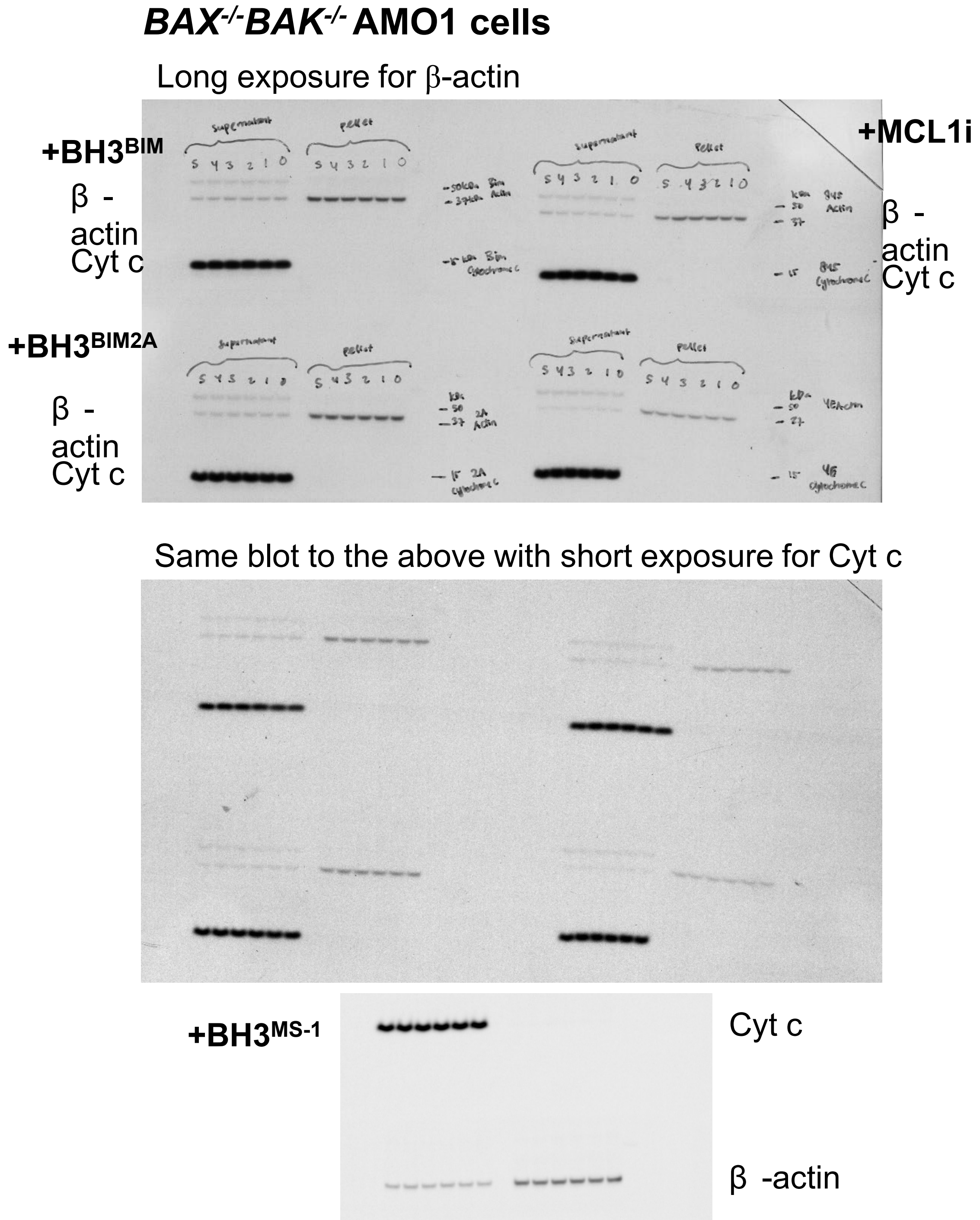
BAX-/-BAK-/- AMO1 cells
Long exposure for β-actin
+BH3^BIM
β - actin
Cyt c
+MCL1i
β - actin
Cyt c
+BH3^BIM2A
β - actin
Cyt c
Same blot to the above with short exposure for Cyt c
+BH3^MS-1
Cyt c
β -actin

Figure 3D

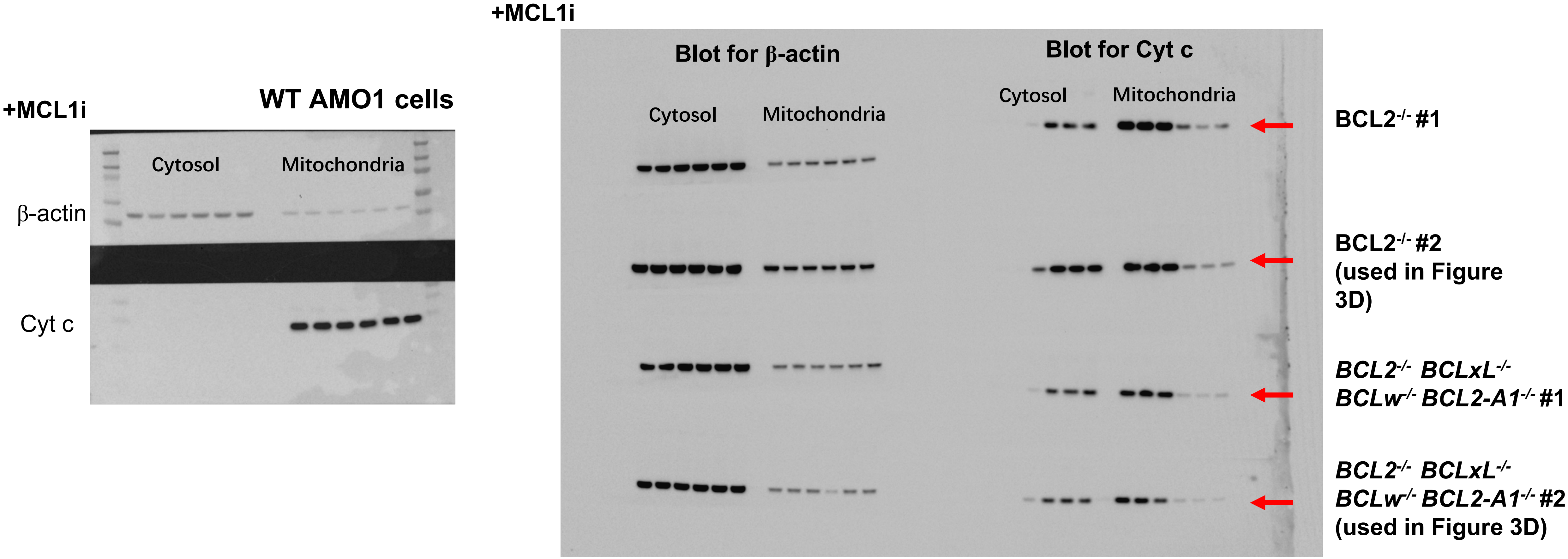
+MCL1i
WT AMO1 cells
Cytosol
Mitochondria
β-actin
Cyt c
+MCL1i
Blot for β-actin
Cytosol
Mitochondria
Blot for Cyt c
Cytosol
Mitochondria
BCL2-/- #1
BCL2-/- #2
(used in Figure 3D)
BCL2-/- BCLxL-/- BCLw-/- BCL2-A1-/- #1
BCL2-/- BCLxL-/- BCLw-/- BCL2-A1-/- #2
(used in Figure 3D)

Figure 7A

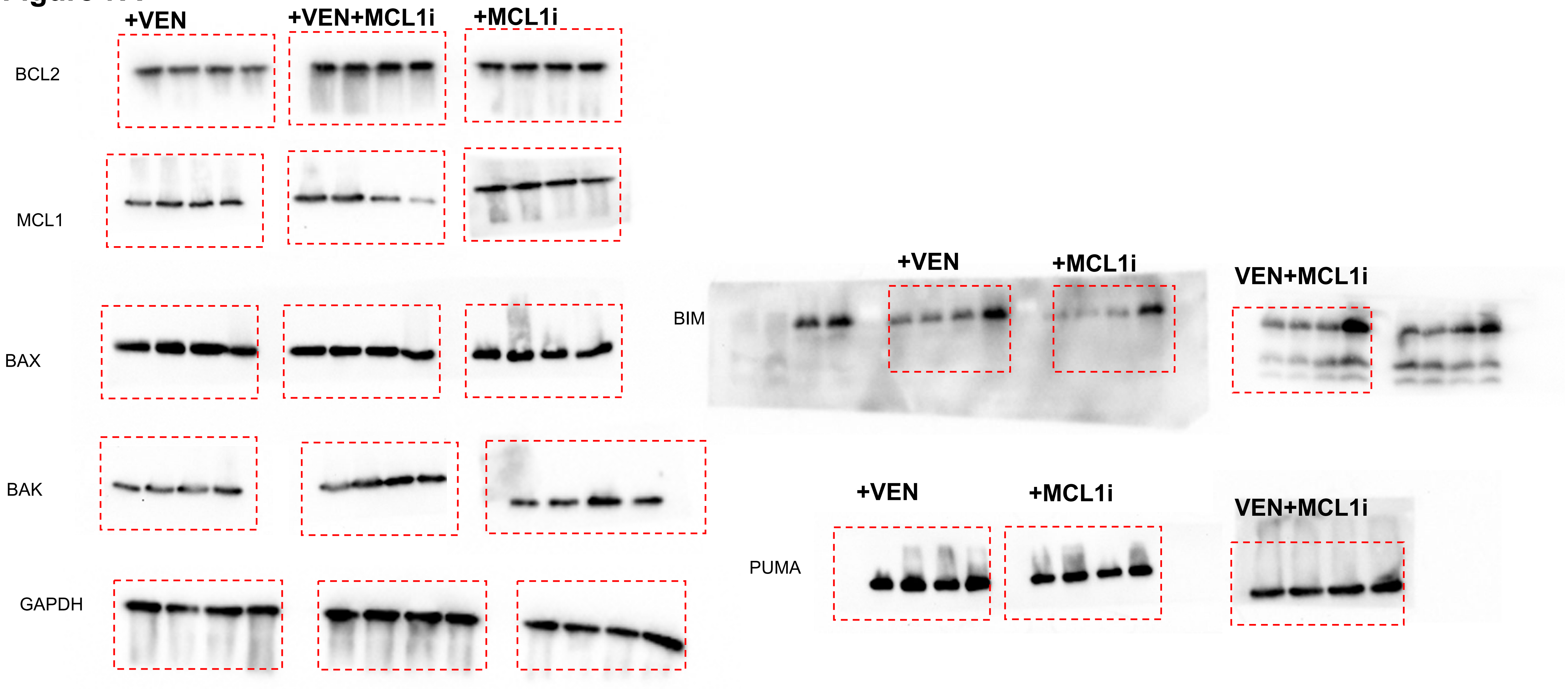
+VEN
+VEN+MCL1i
+MCL1i
BCL2
MCL1
BAX
BAK
GAPDH
BIM
+VEN
+MCL1i
VEN+MCL1i
PUMA
+VEN
+MCL1i
VEN+MCL1i

Figure 7B

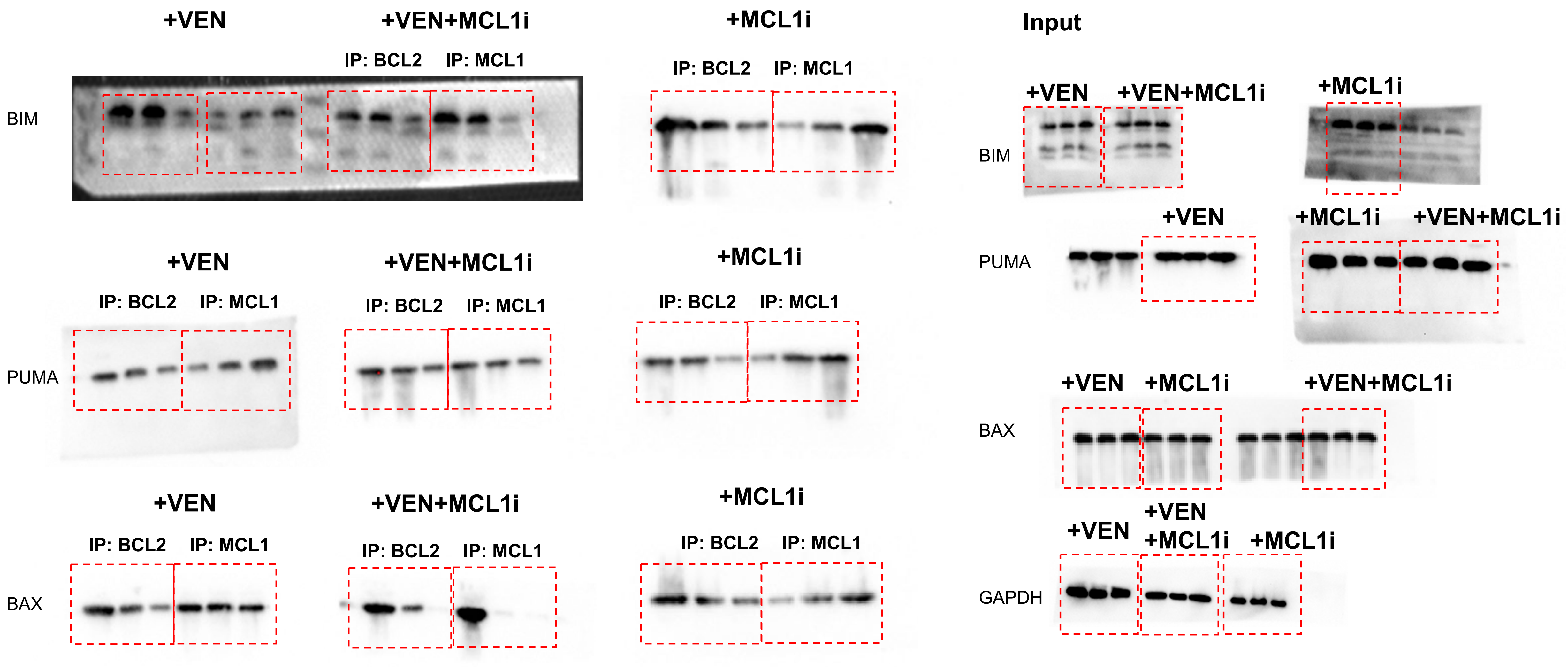
+VEN
+VEN+MCL1i
+MCL1i
Input
IP: BCL2
IP: MCL1
BIM
PUMA
BAX
GAPDH
+VEN
+VEN+MCL1i
+MCL1i
+VEN
+MCL1i
+VEN+MCL1i
+VEN
+MCL1i
+VEN+MCL1i
+VEN
+VEN
+MCL1i
+MCL1i
